# MaxHiC: A robust background correction model to identify biologically relevant chromatin interactions in Hi-C and capture Hi-C experiments

**DOI:** 10.1371/journal.pcbi.1010241

**Published:** 2022-06-24

**Authors:** Hamid Alinejad-Rokny, Rassa Ghavami Modegh, Hamid R. Rabiee, Ehsan Ramezani Sarbandi, Narges Rezaie, Kin Tung Tam, Alistair R. R. Forrest

**Affiliations:** 1 Harry Perkins Institute of Medical Research, QEII Medical Centre and Centre for Medical Research, The University of Western Australia, Perth, Australia; 2 Bio Medical Machine Learning Lab (BML), The Graduate School of Biomedical Engineering, UNSW Sydney, Sydney, Australia; 3 Health Data Analytics Program, AI-enabled Processes (AIP) Research Centre, Macquarie University, Sydney, Australia; 4 Bioinformatics and Computational Biology Lab, Department of Computer Engineering, Sharif University of Technology, Tehran, Iran; 5 Center for Complex Biological Systems, University of California Irvine, Irvine, California, United States of America; University of Michigan, UNITED STATES

## Abstract

Hi-C is a genome-wide chromosome conformation capture technology that detects interactions between pairs of genomic regions and exploits higher order chromatin structures. Conceptually Hi-C data counts interaction frequencies between every position in the genome and every other position. Biologically functional interactions are expected to occur more frequently than transient background and artefactual interactions. To identify biologically relevant interactions, several background models that take biases such as distance, GC content and mappability into account have been proposed. Here we introduce MaxHiC, a background correction tool that deals with these complex biases and robustly identifies statistically significant interactions in both Hi-C and capture Hi-C experiments. MaxHiC uses a negative binomial distribution model and a maximum likelihood technique to correct biases in both Hi-C and capture Hi-C libraries. We systematically benchmark MaxHiC against major Hi-C background correction tools including Hi-C significant interaction callers (SIC) and Hi-C loop callers using published Hi-C, capture Hi-C, and Micro-C datasets. Our results demonstrate that 1) Interacting regions identified by MaxHiC have significantly greater levels of overlap with known regulatory features (e.g. active chromatin histone marks, CTCF binding sites, DNase sensitivity) and also disease-associated genome-wide association SNPs than those identified by currently existing models, 2) the pairs of interacting regions are more likely to be linked by eQTL pairs and 3) more likely to link known regulatory features including known functional enhancer-promoter pairs validated by CRISPRi than any of the existing methods. We also demonstrate that interactions between different genomic region types have distinct distance distributions only revealed by MaxHiC. MaxHiC is publicly available as a python package for the analysis of Hi-C, capture Hi-C and Micro-C data.

## Author summary

MaxHiC is a robust machine learning based tool for identifying significant interacting regions from both Hi-C and capture Hi-C data. All the current existing models are designed for either Hi-C or capture Hi-C data, however we developed MaxHiC to be applicable for both Hi-C and capture Hi-C libraries (two different models have been used for Hi-C and capture Hi-C data). MaxHiC is also able to analyse very deep Hi-C libraries (e.g., Micro-C) without any computational issues. MaxHiC significantly outperforms current existing Hi-C significant interaction callers and even Hi-C loop callers in terms of enrichment of interactions between known regulatory regions as well as biologically relevant interactions.

This is a *PLOS Computational Biology* Methods paper.

## Background

Hi-C is a genome-wide chromosome conformation capture technology used to identify interactions between pairs of genomic regions. Hi-C libraries consist of a pair of reads which can be mapped to the genome for identifying pairs of interacting regions. The data is typically summarized as a contact matrix, in which each row and column correspond to a (fixed-bin) fragment of the genome and each cell shows the number of reads supporting an interaction between the two bins. Hi-C data has multiple biases due both to the multiple steps used in the protocol and the proclivity of closely located genomic regions that are more likely to interact. Not all interactions are biologically relevant and thus the important challenge in the analysis of Hi-C data is to distinguish between background/artefactual interactions and those interactions that are more likely to be functional/regulatory.

There are two main approaches used to identify significant interactions in Hi-C data. The first, as used in Hi-C-DC [1], attempts to quantify known sources of biases (fragment length, GC content and mappability), and use them as variables in a background expectation function. The second approach, as used in Fit-Hi-C [2], CHiCAGO [3] and GOTHiC [4], assumes that all Hi-C biases are reflected in the read count data of interactions. Therefore, the observed or normalised read counts are used to assign a P-value to interactions. In order to account for unknown sources of bias a separate bias parameter is assumed for each bin. The tools using the first approach are limited to analysis of Hi-C libraries generated with restriction enzymes, whereas those using the second approach can also work with DNase Hi-C experiments in which the fragment sizes are unknown.

Rao *et al*. [5] also proposed HiCCUPS, which is not a background correction model, but can be used to identify chromatin loops. The term chromatin loop refers to the interaction between promoters and enhancers, genes or architectural loops, or polycomb-mediated regions [6]. Loop anchors usually happen at domain boundaries and bind CCCTC-binding factor (CTCF) sites. Chromatin loops can be detected as enriched Hi-C interacting regions compared to their neighbourhood regions. HiCCUPS identifies clusters of Hi-C contact matrixes and reports the centroid of the cluster, in which the frequency of the contact matrix is significantly higher than the local background. Using a local background and reporting only the centroid of a cluster results in identification of a small number of significant Hi-C interactions for a small range of distances ~50kb to ~5000kb. Peakachu [7], and Mustache [8] two other loop callers use machine learning techniques to identify chromatin loops. Peakachu builds loop-classifying models by using Random Forest classification techniques to predict chromatin loops from Hi-C data. Mustache uses a new computer vision technique, scale-space theory, to predict blob-shaped objects in Hi-C and Micro-C contact data.

We introduce MaxHiC (Maximum Likelihood estimation for Hi-C), a negative binomial model that uses a maximum likelihood technique to correct the complex combination of known and unknown biases in both Hi-C and capture Hi-C libraries. Benchmarking against current leading background correction models (GOTHiC, CHiCAGO, Fit-Hi-C, Fit-Hi-C2 [9], HiC-DC+ and loop callers (HiCCUPS, Peakachu [7], and Mustache [8].) shows that MaxHiC identifies interactions that contain substantially more evidence of biological relevance. Interacting regions identified by MaxHiC are significantly (P-value < 0.05) enriched for H3K27ac, H3K4me1, CTCF binding sites, DNase hypersensitive sites, and GWAS SNPs. They are also more likely to be annotated as promoters, enhancers, and insulator elements. More importantly the interacting pairs are substantially enriched for interactions between regulatory regions and non-coding polymorphisms linked by eQTL to target genes.

MaxHiC is an open-source python package and is available here https://github.com/bcb-sut/MaxHiC.

## Results

### A negative binomial regression background model for Hi-C data

Here we present MaxHiC, a novel background correction model for identifying statistically significant interacting regions in both general and capture Hi-C libraries. In MaxHiC, the genome is divided into fixed size non-overlapping bins, and the read counts supporting interactions between bins are tested for significance. The read-count of interactions are assumed to follow a negative binomial distribution which is widely used to model over dispersed count data [10, 11]. The mean parameter of the negative binomial distribution for each interaction is calculated as a function of the bias factors of its two ends and their genomic distance.

It is known that the contact frequency between pairs of regions linked by Hi-C reads decays as a function of distance along the chromosome [12]. A large fraction of Hi-C reads represents background ligation products of nearby genomic regions. In MaxHiC, distance is modelled by a function that decreases at increasing genomic distances to reach a small but constant non-zero value to account for background ligations. For *trans*-interactions, we use the same constant value as observed for distant *cis* interactions.

Bias factors of bins are calculated as a function of their total read-count and the mappability of their surrounding bins. All of the parameters of the model are learned by maximizing the logarithm of likelihood of the observed interactions using the ADAM algorithm [13]. The model is trained in multiple iterations; in each iteration, putative interactions with the best P-values are identified and set aside. These significant interactions are ignored in the next phase of training to prevent the trained background model from becoming biased [2]. The same approach is also used in MaxHiC to analyze Capture-Hi-C data. The only difference is that separate sets of parameters are learned for modelling target-target, target-non-target and non-target-non-target interactions to account for the fact that interactions involving one or two of the targeted regions will be enriched to different levels compared to non-targeted regions. A schematic of the MaxHiC model for analysis of general Hi-C is shown in S1 Fig and a detailed explanation of both models is provided in the methods section.

### MaxHiC vs other leading Hi-C tools

In order to evaluate the performance of MaxHiC, we performed a comprehensive benchmarking between MaxHiC and Hi-C significant interactions callers (SIC) including GOTHiC, Fit-Hi-C, Fit-Hi-C2, HiCDC+ [14], and CHiCAGO (for capture Hi-C only). We also compared the performance of MaxHiC with Hi-C loop callers including HiCCUPS [5], Peakachu [7], and Mustache [8]. We used several published Hi-C and capture Hi-C datasets to perform the analyses (see more details about the samples in **S1 Table**).

We first used MaxHiC to identify significant interactions (at 1kb, 5kb and 10kb bin sizes) in two publicly available Hi-C data from Rao *et al*. [5] (on the GM12878 EBV transformed B-lymphoblastoid cell line and human mammary epithelial cells (HMEC)) and one set of capture Hi-C data from Mifsud *et al*. [15] (also on GM12878). For each library we also used the above-mentioned background correction models and loop callers with their default parameters to identify significantly interacting regions. Tables 1 and S2 summarise the numbers of significantly interacting pairs found using each method. Notably the number of interactions called as significant by each method varied greatly (from 10,487 using HiCCUPS, to 8,136,100 using GOTHiC on GM12878).

**Table 1:** Statistical summary of significant interactions identified by MaxHiC, GOTHiC, Fit-Hi-C, Fit-HiC2, HiCDC+, HiCCUPS, Peakachu, and Mustache in the GM12878 Hi-C library from Rao *et al*. [5]. at bin-sizes 5kb and 10kb. Statistical summaries for HMEC library are provided in **S2 Table**, respectively.

1kb			MaxHiC	GOTHiC	Fit-Hi-C	Fit-Hi-C2	HiCDC+	HiCCUPS	Peakachu	Mustache	All
percentage of significant interactions			0.03	9.4	16.6	17.1	0.05	-	-	-	100
average read-count of interactions			18.1	2.9	1.1	1.5	11.8	-	-	-	1.2
min read-count of cis interactions			4	2	1	1	4	-	-	-	1
median distance of cis interactions (kb)			3	14	30832	29426	5	-	-	-	544
**5kb**											
percentage of significant interactions			0.17	9.25	0.8	0.9	0.07	0.0095	0.0172	0.0091	100
average read-count of interactions			21.3	7.85	5.6	5.9	16.7	32.39	28.49	36.16	1.77
min read-count of cis interactions			3	2	1	1	3	11	10	14	1
median distance of cis interactions (kb)			130	85	5235	5014	245	110	145	165	15173
**10kb**											
percentage of significant interactions			0.32	7.32	1.06	1.1	0.11	0.0094	0.0163	0.0066	100
average read-count of interactions			29.4	15.17	12.3	13.5	26.6	58.62	46.37	62.74	2.38
min read-count of interactions			3	2	2	2	5	11	9	16	1
median distance of cis interactions (kb)			270	170	7590	7426	480	150	230	330	6990

* HiCCUPS, Peakachu, and Mustache calls interactions with FDR < 0.05 as significant. For all other methods we used a threshold of P-value < 0.001 to identify significant interactions.

As the p-values from all models may not be comparable, we ranked interactions identified by each tool, based on their reported p-value and then took the top 100K and 20K interactions reported by each tool. S2 and S3 Figs show Venn diagrams of the top 100K and 20K interacting pairs identified by each method at 10kb bin sizes.

**Fig 1:**
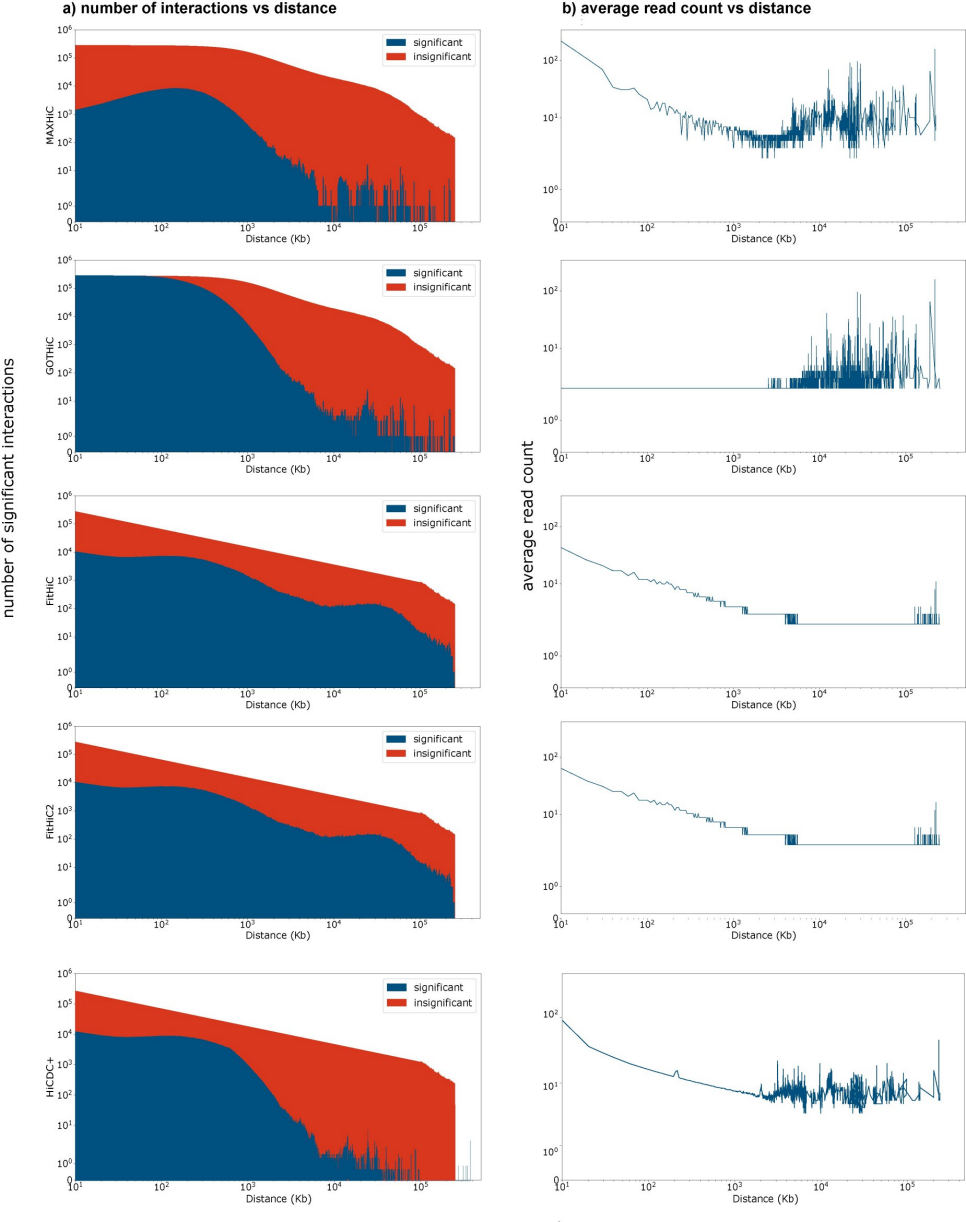
Background modelling as a function of distance in MaxHiC and other HiC tools. **a)** Average read-count of significant (blue) and insignificant interactions (red) at different genomic distances identified by different Hi-C interaction callers at 10kb bin size on the Rao *et al*. [5] GM12878 data. **b)** Number of significant interactions identified by the five Hi-C interaction callers in different genomic distances on Rao *et al*. GM12878 sample at fragment size 10kb.

The sets of interactions called as significant or insignificant by each method (**Fig 1A** for sample GM12878) had distinct distance distributions. At 10kb bin size, GOTHiC and HiCCUPS had the smallest median distances between interacting regions of 170kb, while that for MaxHiC, Fit-Hi-C, Fit-Hi-C2 and HiCDC+ were 270kb, 7,590kb, 7,426kb and 480kb respectively. The low median length observed for GOTHiC is reflected in the observation that it calls almost all putative interactions below 100kb as significant.

Comparing the interactions called as significant by each method revealed interactions identified by MaxHiC had substantially more read support than those identified by all SIC models. Importantly, the number of significant interactions identified by MaxHiC is almost two times more than HiCDC+, however, the average read count of significant interactions in MaxHiC is much higher than HiCDC+ (Tables 1 and S2). Both the average number of reads and minimum number of reads supporting a significant interaction were higher for those called by MaxHiC than other models (Tables 1 and S2).

We also compared the average read count of MaxHiC’s interactions with the loop caller methods. As Tables 1 and S2 show the average read count of MaxHiC’s interactions is smaller than loop callers which was expected as loop callers only report the average signals from selected regions of interest (the centroid of the clusters, CTCF loops, H3K27ac loops, etc), in which the reported frequency for the selected regions is either the total number of interactions in the local cluster or the maximum frequency of all interaction in the local cluster. We then compared ranked interactions identified by MaxHiC based on their reported P-value and compared the average read count of top 20k MaxHiC’s interaction with loop callers at 10kb bin size. Interestingly the average read count of significant interactions for MaxHiC (68.6) was higher than the loop callers (HiCCUPS: 58.6, Peakachu: 46.4, Mustache: 62.7).

By examining the average read count of significant and insignificant interactions called by each SIC method (**Fig 1B** for GM12878), it is revealed that at all distances, significant interactions called by MaxHiC and HiCDC+ had substantially more reads than those called as insignificant. GOTHiC only demonstrated this trend at distances above 100kb. At all distances, paired regions called as significantly interacting by MaxHiC had higher average read count support than the other five methods. This was also reflected in the minimum number and average number of reads required to call an interaction as significant (S4 Fig). We observed a similar pattern when considering interactions calculated using different fragment sizes (S5 Fig shows the same plots using 1kb, 5kb, 10kb, and 50kb bin sizes).

### Evidence of regulatory potential for interacting regions identified by MaxHiC

**Fig 2:**
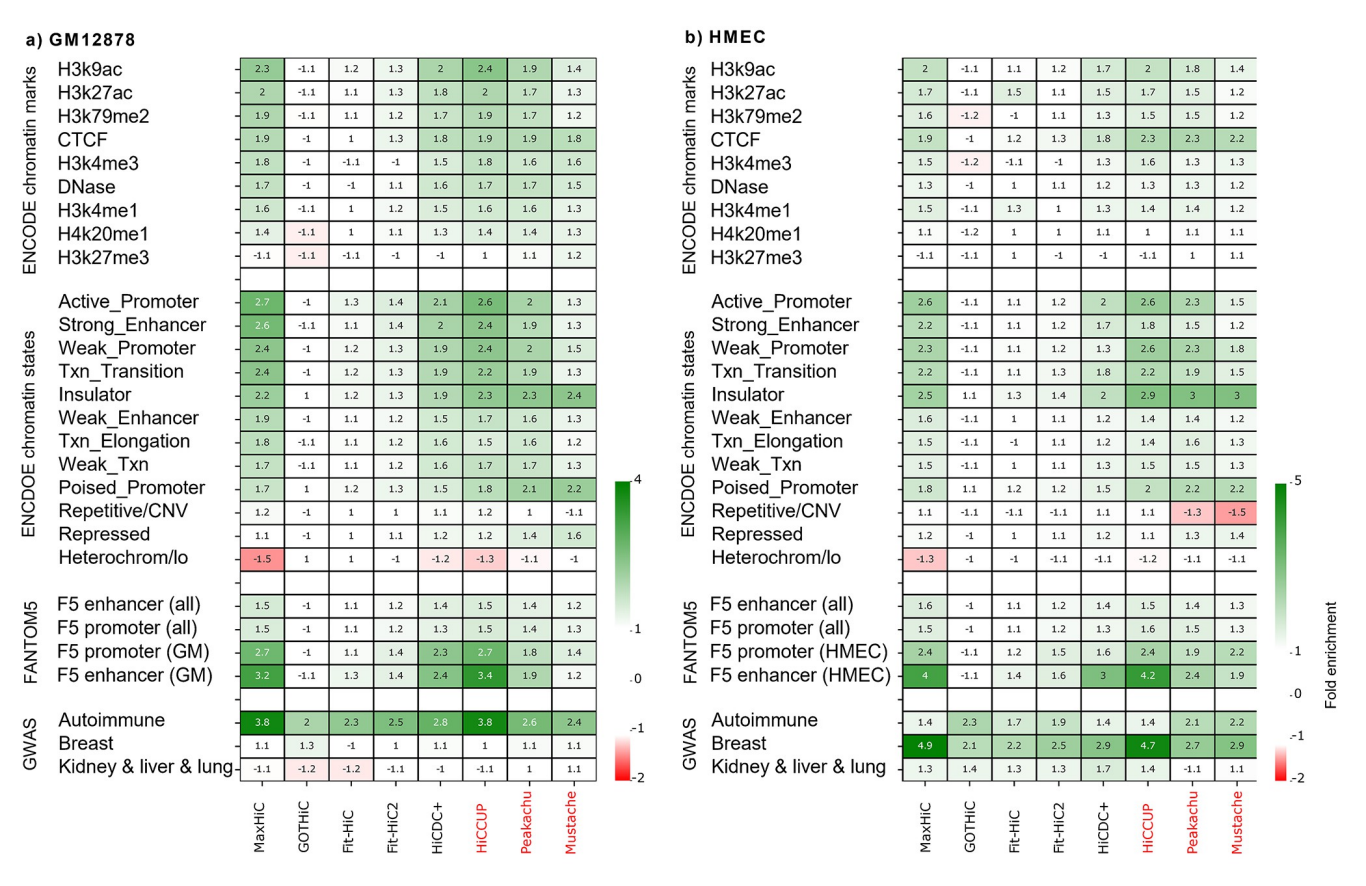
Enrichment of regulatory potential in regions identified by MaxHiC and other tools. **a)** Enrichment of GM12878 specific histone marks, putative regulatory regions and GWAS signal in regions identified by MaxHiC in the Rao *et al*. [5] GM12878 dataset. **b)** As in (a), but enrichment of signals in the HMEC dataset are shown. ENCODE chromatin states are from GM12878 and HMEC data respectively. For FANTOM5, enrichment is shown for all FANTOM5 promoters and enhancers and for those only active in GM12878 and HMEC. GWAS SNPs for autoimmune disease, breast and kidney/liver/lung disorders (**S4 Table**) were downloaded from Maurano *et al*. [29]. Note: HiCCUPS calls interactions with FDR < 0.05 as significant. For all other methods we used a threshold of P-value < 0.001 to identify significant interactions.

We next investigated whether interacting regions identified by each method as significant were enriched for genomic and epigenetic annotations indicative of regulatory regions. Comparison of the interacting regions with histone mark profiles generated for GM12878 and HMEC by the ENCODE consortium [16–18] revealed the regions identified by MaxHiC were significantly (P-value < 0.05) enriched for H3K9ac, H3K27ac, H3K79me2, H3K4me3, H3K4me1, and H4K20me1 marks (**Fig 2**). They were also enriched for DNAse hypersensitive sites and CTCF binding but not for the repressive mark H3K27me3. Importantly there was very little if any enrichment of these features in regions called as significant by GOTHiC, Fit-Hi-C, and Fit-Hi-C2. There were also enrichments for HiCDC+’s regions, however, these enrichments were less substantial than MaxHiC’s regions. Interestingly MaxHiC’s regions had higher enrichments than regions identified by the loop callers Peakachu and Mustache but had a similar level of enrichment with HiCCUPS. Notably, MaxHiC identified 34-fold more interactions.

We also used MaxHiC to investigate the enrichment of other important histone markers, such as H3K4me1, H3K4me2, H3K4me3, H3K27me3, H3K9me3 in the significant interactions. As **S6 Fig** shows all histone marks used in the analysis, were significantly enriched (P-value < 0.05) for the significant interactions identified in GM12878, except H3K27me3, H3K9me3, and H4K20me1.

To ensure that MaxHiC’s performance is not affected by the number of statistically significant chromatin interactions used in the analyses, we ranked interactions identified by each tool, based on the reported p-value, and then took the top 10K, 20K, 50K, 100K interactions reported by each tool. We then repeated the enrichment analysis. As S7 Fig shows MaxHiC’s interactions have the highest enrichment for regulatory features compared to all other tools.

For the GM12878 analysis we also observed significant enrichment (P-value < 0.05) of transcription factor binding sites from ENCODE ChIP-seq data (S8 Fig and S3 Table). Again, MaxHiC had much higher enrichment than SIC models and the loop callers Peakachu, and Mustache while it had a similar level of enrichment with HiCCUPS for GM12878 specific transcription factor binding sites. Together this suggests that both MaxHiC and HiCCUPS identify interactions between regions with regulatory potential however MaxHiC identifies substantially more (34-fold).

Next, the interacting regions identified by MaxHiC were significantly enriched (P-value < 0.05) for ENCODE annotated enhancers, promoters, insulators and transcription states [19, 20] (**Figs 2**, and **S7** for top 10k, 20k, 50k, 100kinteractions analyses). They were also significantly depleted (P-value < 0.05) of regions annotated as heterochromatin. Again, HiCCUPS showed similar levels of enrichment while Fit-Hi-C and Fit-Hi-C2, HiCDC+ showed some lower level of enrichment for some of the chromatin states.

Besides the regulatory regions predicted by ENCODE, significant interactions identified by MaxHiC also contained a much higher fraction of promoters and enhancers identified by FANTOM5 as active in GM12878 and HMEC (in comparison to all FANTOM5 promoters and enhancers) (**Fig 2**) indicating that MaxHiC not only enriches for promoters and enhancers but those active in the cell type from which the Hi-C data was generated.

Importantly, interacting regions identified by MaxHiC and HiCCUPS on the GM12878 dataset were significantly (P-value < 0.05) more likely to overlap autoimmune SNPs identified in GWAS while those identified in the HMEC were significantly (P-value < 0.05) more likely to overlap breast trait associated SNPs (**Fig 2,** GWAS traits considered are provided in S4 Table). No such enrichment was observed for SNPs associated with unrelated traits such as kidney, lung, and liver traits.

Lastly, we compared interactions found by MaxHiC to those from the next top SIC method Fit-HiC2 (based on top 10k, 20k, 50k, and 100k interactions analyses) and the top loop caller method, HiCCUPS. In the comparison to Fit-Hi-C2, the interactions found by MaxHiC alone were significantly enriched (P-value < 0.05) for interactions between regulatory regions whereas those found by Fit-Hi-C2 alone showed no enrichment and were near the background expectation of 1 (S9A Fig). In contrast the MaxHiC and HiCCUPS -specific interactions were enriched to similar levels (S9B Fig).

S10 Fig shows an example of a Hi-C interaction identified by MaxHiC as significant, which was not identified as a significant interaction by other tools. Both sides of the interaction have strong signals of regulatory features. (S10 Fig).

### MaxHiC enriches for interactions between regulatory regions

**Fig 3:**
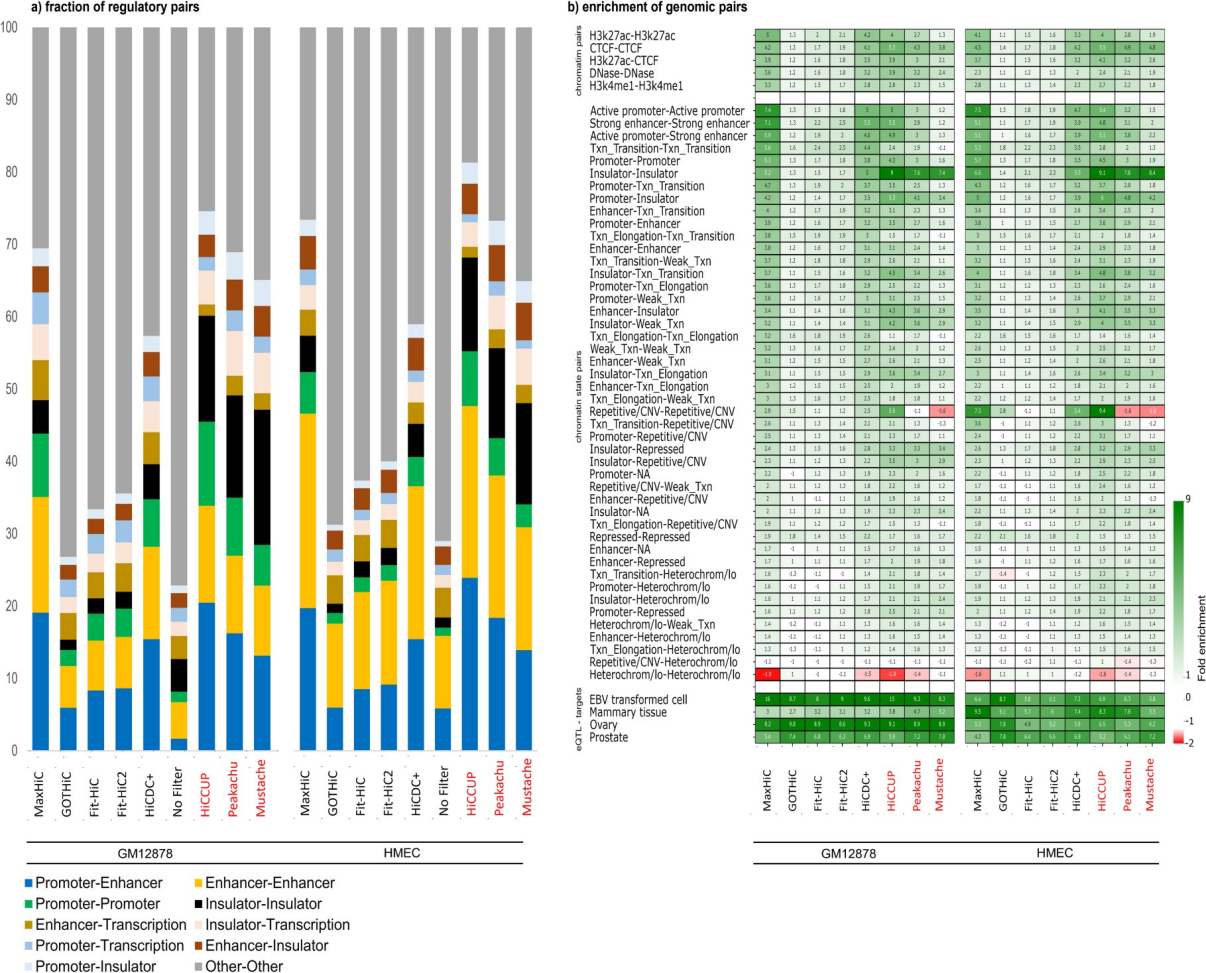
MaxHiC enriches for interactions between regulatory regions, eQTL-target pairs and known enhancer-promoter pairs. **a)** Fraction of regulatory elements in the significant interactions identified by the four models. **b)** Enrichment of cell line specific chromatin pairs, chromatin state pairs, and eQTL pairs that overlapped with both ends of the significant interactions identified by the six models at fragment size 10kb. The enrichment is calculated by dividing the fraction of features overlapping both ends of the significant interactions identified by each model by the fraction of features overlapping both ends of all interactions (*see methods*).

We next examined the fractions of interacting pairs identified by each method involving regulatory elements at both ends. We used GM12878 related ChromHMM predicted chromatin segments from ENCODE. We specifically focused on promoter-enhancer, promoter-promoter, enhancer-enhancer and insulator-insulator pairs. In the GM12878 dataset approximately 12% of the raw unfiltered pairs fell into at least one of the above categories (**Fig 3A**). Strikingly, the significant interactions identified by MaxHiC were almost 4 times (47%) more likely to fall into one of these four categories (**Fig 3A**). Notably ~17% of the pairs corresponded to promoter-enhancer interactions, ~15% enhancer-enhancer, ~8% promoter-promoter and ~7% to insulator-insulator interactions. Similar levels of enrichment were observed for HiCCUPS and Peakachu but as noted above, HiCCUPS identified almost 34-fold fewer significant loops. HiCDC+’s interactions were also significantly enriched for promoter-enhancer, promoter-promoter, enhancer-enhancer and insulator-insulator pairs. In contrast, the other methods had distributions similar to the unfiltered pairs with Fit-Hi-C, Fit-Hi-C2 and GOTHiC showing fractions slightly above that of the unfiltered pairs. Extending this to any interaction involving an enhancer, promoter or insulator increased the fraction of annotated pairs to 70%. Similar fractions were observed with the HMEC data.

We also repeated this analysis for the top 100K and 20K interactions identified by each model. Interestingly, for the top 100K interactions, MaxHiC had higher enrichment than all SIC models and loop callers in both GM1278 and HMEC cell lines. This enrichment was much higher in the top 20K interactions analysis (S11 Fig). In the top 20K interactions on HMEC cell line, 99%) of MaxHiC’s interactions were annotated as enhancers, promoters or insulators.

**Fig 3B** shows the relative enrichment for interactions involving pairs of chromatin features and predicted chromatin states. Similar to **Fig 2** we observed enrichment of interactions involving active chromatin states and depletion of heterochromatin-heterochromatin pairs using the interactions called as significant by MaxHiC and HiCCUPS and to a much lesser extent with Fit-Hi-C and Fit-Hi-C2. HiCDC+ also had a significant level of enrichment for interactions involving pairs of chromatin features, but less compared to MaxHiC.

We next used expression quantitative trait loci (eQTL) pairs, identified in EBV-transformed lymphocytes as part of the Genotype-Tissue Expression (GTEx) Project [21] to examine enrichment of eQTL SNPs in regions that interact with promoters. Approximately 2.1% of the interactions identified by MaxHiC in the GM12878 cell line overlapped at least one eQTL pair. This corresponded to a 15.7-fold enrichment (**Fig 3B**). In contrast GOTHiC, Fit-Hi-C’, Fit-Hi-C2, and HiCDC+’s interactions were lower (8.3, 7.9, 9, and 9.6-fold enriched respectively). Interestingly, the enrichment of eQTL pairs in MaxHiC’s interaction was slightly higher than HiCCUPS and much higher than Peakachu and Mustache. Matching analyses on HMEC confirmed similar enrichments for regulatory features and eQTLs from mammary tissue (**Fig 3B**).

### Genomic spacing of interacting regulatory elements

**Fig 4:**
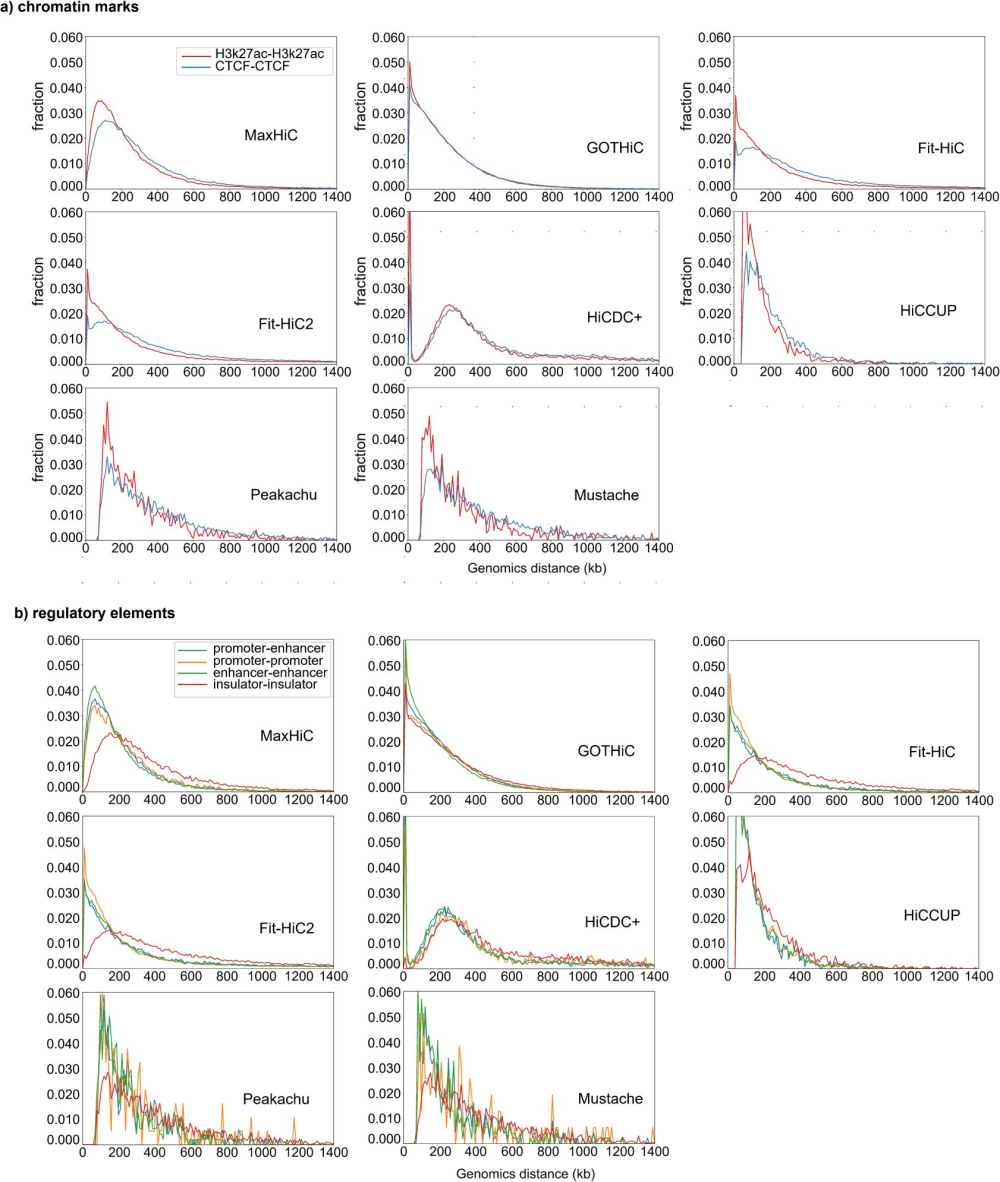
Distance distribution for interactions between different classes of regulatory elements. **a)** Comparison of spacing observed for H3k27ac-H3k27ac and CTCF-CTCF structural loops in the interactions identified by MaxHiC in the Rao *et al*. GM12878 dataset. **b)** Comparison of spacing observed for promoter-promoter, enhancer-promoter, enhancer-enhancer and insulator-insulator pairs in the interactions identified by MaxHiC in the Rao *et al*. [5] GM12878 dataset. Plots use fragment bin-size 5kb and window size 5kb.

Using the putative regulatory region annotations from the analysis above, we next examined the spacing preferences for interactions between different regulatory features. We first examined preferred spacing for H3K27ac-H3K27ac and CTCF-CTCF structural loops. For MaxHiC, Fit-Hi-C, and Fit-Hi-C2 we observed the spacing between CTCF pairs to be larger (130kb) than H3K27ac-H3K27ac pairs (80kb) (**Fig 4A**). In both cases there were long tails reaching beyond 1Mb. Performing the same analysis on annotated regulatory regions revealed promoter-promoter, enhancer-promoter, and enhancer-enhancer pairs had almost identical spacing preferences (with median spacings of 70kb, 70kb and 90kb respectively), while insulator-insulator pairs were considerably further apart with a median spacing of 170kb (**Fig 4B**). We did not see this trend for HiCDC+ and loop callers, however we note HiCCUPS did not identify significant interactions below 50kb and above 5Mb.

### MaxHiC contact maps and aggregate peak analyses

**Fig 5:**
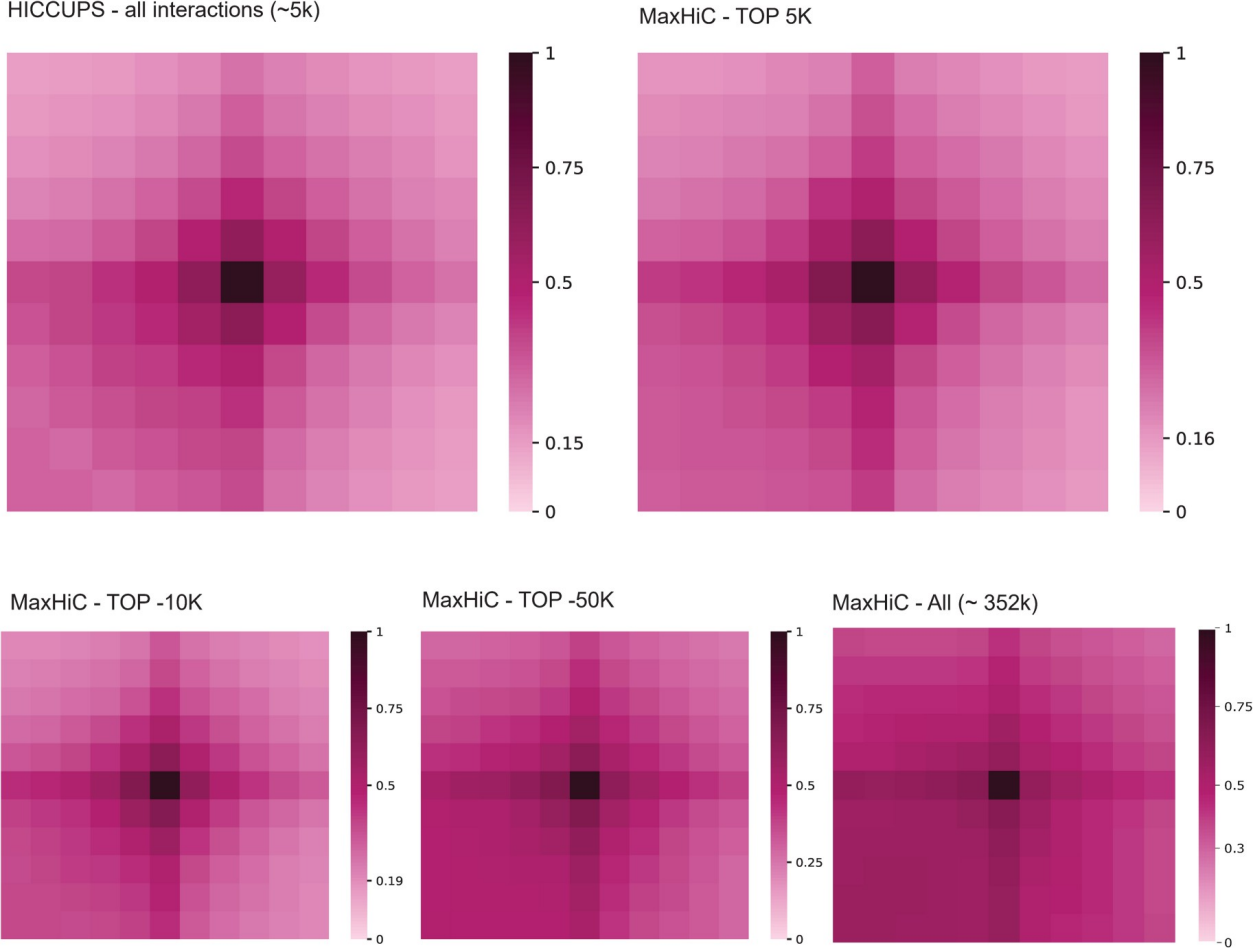
Aggregate peak analysis (APA) of significant interactions called by MaxHiC and HiCCUPS. Interactions were called at 10kb resolution using the GM12878 Hi-C dataset from Rao *et al*. The analysis shows the aggregate background interaction frequency of 5 bins upstream and downstream of the significantly interacting bins. For HiCCUPS the bins at the centroid of the loop regions are used. For HiCCUPS the APA for all 5,348 interactions is shown. For MaxHiC, APA plots are shown for the top 5k, 10k, 50k and all 243k significant interactions.

Comparing contact maps of significant interactions found for MaxHiC and HiCCUPS models (S12 Fig) shows at first glance they are very similar, however in a zoomed in region we can see additional interactions called by MaxHiC that are missed by HiCCUPS. Examining this in more detail revealed as approximately 8-fold more bases are identified as significantly interacting by MaxHiC than HiCCUPS (S13 Fig). However, to further show that these interactions are not random we provide an aggregate peak analysis (APA) comparing signals surrounding significant peaks called by MaxHiC and HiCCUPS. **Fig 5** shows the aggregate background interaction frequency of 5 bins upstream and downstream of the significantly interacting bins. APA plots are shown for the top 5k, and 10k. As the figure shows, MaxHiC and HiCCUPS have almost the same APA patterns and confirmed that the significant interactions identified by MaxHiC are not random and that even when all 352k significant interactions identified by MaxHiC are considered there is strong focal enrichment in the APA. To demonstrate application of MaxHiC to a very large dataset we also analysed a Micro-C dataset [22] consisting of ~480M raw valid interactions. The contact map for this dataset is shown in **S14A Fig**. Reassuringly matching aggregate peak analysis plots confirm there is strong focal enrichment for these significant interactions **S14B Fig**.

### Application of MaxHiC to identify differentially interacting regions

**Fig 6:**
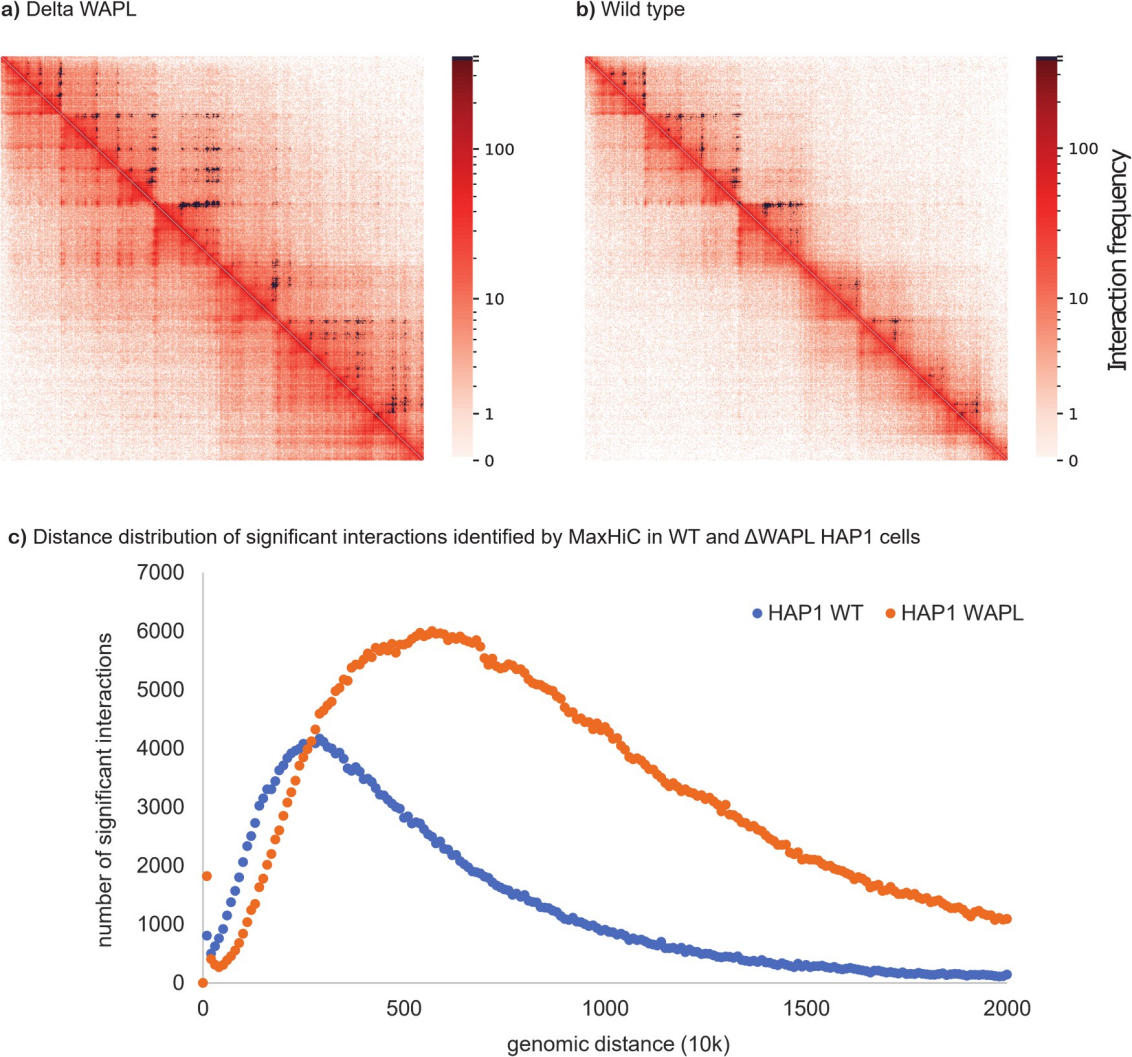
WAPL restricts chromatin loop extension. HiC Contact map comparing significant interactions identified in WT (**a**) and ΔWAPL HAP1 (**b**) cells at 10kb fragment size. Black pixels are significant interactions identified by MaxHiC at P-value <0.001. (chr7: 25M-31M). **c)** Distance distribution of significant interactions identified by MaxHiC (P-value <0.001, 10kb bins) in WT and ΔWAPL HAP1 cells. The number of significant interactions identified in HAP1 WT is 283,028 and the number of significant interactions identified in HAP1 WAPL is 761,820.

To further demonstrate the use of MaxHiC in comparative Hi-C experiments we applied it to Hi-C datasets from cells depleted of the cohesion release factor WAPL (Haarhuis *et al*. [23]) and the polycomb protein RING1B cells (Boyle *et al*. [24]); and their matched wildtype controls. In the case of WAPL-depleted HAP1 cells MaxHiC identified almost 3 times more significant interactions than in matched wildtype cells (**Fig 6A and 6B**). Furthermore the distance distribution of interacting genomic regions found in the ΔWAPL cells (median = 6.4Mb) was significantly longer in comparison to wildtype (median = 3.1Mb) (**Fig 6C**); this reiterates the finding of Haarhuis *et al*. [23].

In the case of the RING1B depleted cells, MaxHiC was able to re-identify differentially interacting regions identified in the original paper (**S15A and S15B Fig** replicate Fig 5A from Boyle *et al*. [24]). We also observed that wildtype cells have a slightly larger median distance between interactions (325kb for WT) than the knockout cells (258kb for RING1B-/-) (**S15C Fig**). Lastly we examined enrichment for repressive H3K27me3 and RING1B sites (using ChIP-seq data from wildtype cells [25]) in interacting regions found in wild type cells and RING1B-/- cells. In all cases the enrichment was close to the genome wide background expectation of 1, however the interactions found in the knockout were slightly more enriched than the wildtype, which suggests the sites are more open for interactions after removal of RING1B (**S15D Fig**).

### MaxHiC identifies more validated pairs of element-gene pairs tested by CRISPRi

Lastly, as significant interactions identified by MaxHiC were enriched for enhancer-promoter interactions we sought to test whether these interactions are enriched for functional regulatory potential. To do this we asked whether interactions identified by MaxHiC and alternative tools run on a K562 Hi-C dataset from Rao *et al*. [5] could connect a set of 134 functional regulatory element-target gene pairs (involving 103 CRISPRi targeted regions) identified in K562 cells [26]. 102 of the 103 CRISPRi targeted regions and 57 of the 134 element-target gene pairs are present in the raw Hi-C data (182M total interactions). MaxHiC identified approximately 275K significant interactions, amongst which 16% (21 of 134) of ​the CRISPRi element-target gene pairs were captured (**S16 Fig**). The next best performing tool Fit-HiC2 only identified 10 of 134 pairs, HiCCUPS identified 1 and Mustache, HiCDC+ and Peakachu failed to identify any of the pairs. From this we conclude that MaxHiC identifies functionally relevant regulatory interactions.

## Discussion

Enhancers have important roles in controlling gene expression. Linking distal regulatory regions such as enhancers to the genes that they regulate is critical to the interpretation of regulatory variants identified in whole genome data. Importantly, genomic variants in enhancers and regulatory elements have been linked to human complex diseases [27, 28]. Therefore, pairing enhancer regions to their regulatory targets is crucial for diagnosis of genetic diseases. Compared to simplistic assignment of enhancers to their nearest promoters, computational methods have improved the prediction of enhancer-promoter pairs [29–32]. However, physical interaction data is still far more convincing in validating the pairs. Hi-C data offers one approach to identify these links, however Hi-C sequencing libraries are extremely noisy because of self-ligations, background ligation artifacts and other complex sources of biases such as GC content and mappability of the sequences. Given this, accurate identification of significantly interacting regions is dramatically affected by the tool used. Here, we have presented MaxHiC, a new tool for identifying significantly interacting regions from Hi-C data (and capture Hi-C data see **S17 and S18 Figs and S5 Table**). Through a dramatically improved discrimination of biologically relevant interactions from background, we showed that MaxHiC enriches for interacting regions with substantially more evidence of regulatory potential (**Figs 1 and 2**) than those identified by existing Hi-C analysis tools [2–4, 9]. This is a critical advance as enhancer-promoter interaction maps are needed to interpret the impact of non-coding variants identified in intergenic regions. Importantly CRISPRi data were able to confirm that MaxHiC enriches for functional enhancer-promoter relationships.

From our analyses, only MaxHiC and HiCDC+ (and to a much lesser extent Fit-Hi-C2) enrich for interactions between regulatory regions, but MaxHiC regions, had much higher enrichment than HiCDC+. Specifically, interactions identified by MaxHiC were enriched for regulatory features, disease-associated genome-wide association SNPs, eQTL pairs and experimentally validated enhancer-promoter interactions. Although interacting regions identified by loop callers had similar levels of enrichment MaxHiC found 34-fold more significantly interacting regions. Our top‘k’ (k:10k, 20k, 50k, and 100k) interactions analyses showed that MaxHiC significantly outperformed not only SIC models, but also loop callers. Additionally, loop callers such as HiCCUPS appears to have limitations on the distances at which interactions can be found and the bin sizes possible. They also need very deep Hi-C resolution and substantial computational resources. From this observation we conclude that MaxHiC is a substantially better tool than existing Hi-C analysis packages.

We also implemented a capture version of MaxHiC to analyse capture Hi-C experiments; in the capture Hi-C technology, target regions (also called baits) are sequenced with higher depth than the others, resulting in three different types of interactions target-target, target-other and other-other with different properties. Capture Hi-C baits, but not other ends, have a further source of bias associated with uneven capture efficiency, thus for capture Hi-C experiments we model each class separately. As **S17 and S18 Figs** show the capture version of MaxHiC also identifies target-interacting regions that are enriched for regulatory features and disease-associated GWAS SNPs. The levels of enrichment are much higher than the regions identified by GOTHiC, Fit-Hi-C, and Fit-Hi-C2, and HiCDC+. The average read count of significant interacting regions identified by MaxHiC were much higher than other models. Importantly, MaxHiC’s interacting regions had higher level of enrichment than the interacting regions identified by CHiCAGO, a specific model designed for the analysis of capture Hi-C data.

In conclusion, MaxHiC is a new open source tool for identifying significant interacting regions from Hi-C and capture Hi-C data. We have demonstrated that it significantly outperforms existing tools in terms of enrichment of interactions between known regulatory regions. We will continue to develop MaxHiC related tools for identifying topologically associating domains (TADs) and for identifying interactions that are significantly different between conditions. As single-cell Hi-C data becomes more broadly available we will also adapt MaxHiC to work with these datasets. Lastly, we believe that with minor modifications the same principles used here to analyse Hi-C data may be more broadly extended to identify significant interactions from RNA-chromatin interaction datasets [33, 34].

### Online methods

#### Tool availability

The MaxHiC source code and python package, a sample dataset, and instructions on how to run MaxHiC are provided at https://github.com/bcb-sut/MaxHiC. For more details about each parameter, please visit the GitHub page. A tutorial on using the MaxHiC package is also provided in **S1 Information** (instructions for MaxHiC). MaxHiC is memory and speed optimized and will work with both single core and multicore machines. It can work with very large Hi-C data sets (as bam file or contact maps generated by other tools) to identify significant interactions at a broad range of resolutions (1kb-500kb).

### Publicly available Hi-C data and data access

Samples GM12878 B-lymphoblastoid cell line, Human Mammary Epithelial cell line, and k562 cell line Hi-C data from Rao *et al*. [5], capture Hi-C sample GM12878 B-Lymphoblastoid from Mifsud *et al*. [15] and mouse cortex Hi-C sample from Dixon *et al*. [35] were used to benchmark MaxHiC against other models. All samples are publically available. More details about the samples are provided in **S1 Table.**

### Mapping, filtering and Interaction calling

MaxHiC takes as input either bam files or contact map files generated by packages such as HiCUP [36] and HiC-Pro [37]. For the analyses presented here, FASTQ paired-end reads were aligned to the human hg19 genome and filtered through the HiC-Pro pipeline (statistics are presented in **S1 Table**). To remove duplicate reads, filter for valid interactions, and generate Hi-C interaction matrices (5kb and 10kb bin-sizes), we set the following parameters: MIN_FRAG_SIZE = 230; MAX_FRAG_SIZE = 1100; MIN_INSERT_SIZE = 120; and MAX_INSERT_SIZE = 990. All experimental artifacts, such as circularized reads and re-ligations, singletons have been filtered out using HiC-Pro and MHiC [38]. We also removed all self-interactions from the downstream analyses. In-house scripts were then used for statistical evaluation of Hi-C libraries (see **S1 Table**).

### The MaxHiC algorithm

In the following, we explain MaxHiC algorithm and mathematics behind the model for the analysis of both Hi-C and capture Hi-C experiments.

### A negative binomial regression background model for Hi-C data

In MaxHiC, a negative binomial distribution has been used to model the read count of interactions based on the bias factors of the two ends and their genomic distance. The negative binomial distribution is the analytic form of a Poisson distribution with its mean parameter following a gamma distribution to account for over-dispersion in data. As shown in Eq 1, the observed read count of interactions, *x*_*ij*_, is modelled using their expected read count, *μ*_*ij*_, and a shared dispersion factor, *r*, between all interactions.


$$x_{ij}∼NB(\mu_{ij},r)$$ (1)

MaxHiC considers ‘neighborhooding’ (similar to local background) when identifying significant interactions. The parameters of the model are learned using a maximum likelihood approach. Gradient descent has been used to find the values of the parameters maximizing the logarithm of the likelihood function. In order to have higher accuracy, by having a higher difference between the read count of meaningful and meaningless interactions, and also to have higher speed, the proposed model uses the interactions between DNA bins, not DNA fragments. DNA is divided into pieces and all reads recorded for each fragment are assigned to the bin containing their middle points. All of the recorded interactions between pairs of bins with at least one read are the input samples used for training the model. After the model has been trained, the P-value of each interaction can be calculated based on the value of reliability, *1—CDF*, for its observed read count.

### Calculating the expected value for the read count

The expected read count for the interaction between bins *i* and *j* is calculated according to Eq 2. *f*(*d*_*ij*_) indicates the expected value of read count to be recorded in the experiment for any two loci with distance *d*_*ij*_, *v*_*i*_ and *v*_*j*_ are bias factors of the two interacting bins. As mentioned above, bins have different characteristics and different probabilities of showing up in the experiment that affects the expected value of their interactions. It’s assumed that the effects of two bins are independent from each other, so the total bias factor has been calculated as a product of the bias factors of the two ends.


$$\mu_{ij}=v_{i}\times v_{j}\times f(d_{ij})$$ (2)

### Calculating bias factors for bins

In order to be applicable to all types of Hi-C protocols, accounting for the unknown sources of bias and also the relation between the known sources, the second approach, mentioned in the introduction, has been adopted in this study. A uniform bias factor has been assumed for each bin, which becomes more valid as the resolution of the analysis increases and the length of bins become shorter. We expect the bins included in more interactions with higher reads to have higher tendency to show up in the experiment. In addition, the total number of interactions that a bin has been included in, would have no effect on its bias. To deal with these problems, the bias factor for bins have been calculated using Eq 3 in which *v*_*base*_, *b* and *b*_*m*_ are the parameters of the function and *s*_*i*_ is the total number of reads observed for bin *i*. Here it is assumed that the total number of reads observed for each bin is a measure of its ability to show up in the final results of the test, and bias factor is assumed to be a linear function of *s*_*i*_ in log-log space. *v*_*base*_ is also considered as the base possible value for the bias factor. This prevents the bins with very few recorded reads to affect the parameters in the training process and it also prevents their related interactions with very few reads from showing up as significant because of their abnormally low bias. Many tools do this by putting a threshold and filtering bins with less bias, but in MaxHiC it is done automatically without any need for adjusting the threshold. As illustrated in Eq 3, *v*_*i*_ is proportional to *s*_*i*_ raised to a power *b*. The power can be learned and if it becomes less than one it can express a saturation phenomena. As duplicated read pairs are discarded in post-processing, having more read count for two interacting bins as the read count of their interaction increases, becomes lower than their initial potential.


$$v_{i}=maximum(e^{b\times \ln\left(s_{i}\right)+b_{m}},v_{base})\Rightarrow v_{i}=maximum(s_{i}^{b}\times e^{b_{m}},v_{base})$$ (3)

### Calculating expected read count as a function of distance

The expected read count as a function of distance for cis interactions is illustrated in Eq 4. The function is the maximum of two values. The first one, *f*^*v*^(*d*_*ij*_), is a degree 3 polynomial function of genomic distance in log-log space which specifies the expected read count of interactions based on the linear genomic distance between the two interacting loci. The reason for using an exponential function is the fact that Brownian motion and diffusion can be modelled using an exponential function. This assumption is also consistent with the power law function observed for the read count as a function of distance which has been introduced by Lieberman *et al*. for the first time [39]. The degree of 3 has been used so the function can have varying curvature in different distances. In this function, the logarithm of distance has been used instead of the distance itself, as DNA is not a straight string and has multiple folding with different scales that causes proximity to have a closer relation with the logarithm of the length than the length itself. A similar concept has been used in Fit-Hi-C [2], CHiCAGO [3] and HiC-DC [1] methods. The biggest difference is the fact that all of these methods consider a strictly decreasing function for the expected read count which approaches zero as the genomic distance increases and causes all interactions between far genomic loci to be considered significant while they may have only one read. This cannot be completely logical as farther than some specific distance, random background collisions occur due to three dimensional closeness not the genomic closeness. This three dimensional closeness can be due to closeness to the fixed three dimensional structures such as loops or random dynamic crossing of DNA string while two different parts of it are having contact. The expected read count for these kinds of random interactions has been modeled using a constant parameter, *f*^*c*^. Therefore, in close genomic distances $f^{v(d_{ij})}$ is dominating while the value of $f^{v(d_{ij})}$ becomes very small, the value of *f*^*c*^ dominates the expected read count. For trans-interactions, similar to the cis interactions for far loci, a constant parameter has been used to denote the expected value of read count for any random background trans-interactions.


$$f(d_{ij})=max(f^{v(d_{ij})},f^{c})$$ (4)

$$f^{v(d_{ij})}=e^{\left(a_{3}.{\ln\left(d\right)}^{3}+a_{2}.{\ln\left(d\right)}^{2}+a_{1}.\ln\left(d\right)+a_{0}\right)}$$

Maximum function is not a smooth function to use in optimization. Therefore, instead of maximum, the soft-maximum function has been used. This soft-maximum resembles the maximum function. When an argument, for example *α*, is larger than the other, it’s exponential becomes far more larger and so it’s related factor, $\gamma_{\alpha }^{\tau }$, approaches 1 while the other factor, $\gamma_{\beta }^{\tau }$, approaches 0. Therefore, the final value will be approximately equal to *α* itself.


$$\tau =softmax(\alpha ,\beta )⟹\tau =\gamma_{\alpha }^{\tau }\times \alpha +\gamma_{\beta }^{\tau }\times \beta$$ (5)

$$\gamma_{\alpha }^{\tau }=\frac{e^{k\times \alpha }}{e^{k\times \alpha }+e^{k\times \beta }},\gamma_{\beta }^{\tau }=\frac{e^{k\times \beta }}{e^{k\times \alpha }+e^{k\times \beta }}$$

### Training the model in multiple rounds

As mentioned above, the method models read count of interactions occurring based on Brownian motion, not the meaningful chromatin contacts, which are called real interactions from now on. But real interactions are also among the data and can affect the parameters of the model and pull them up. For example, in distances where there are more read interactions, the expectation may become higher than other distances. To deal with this bias, the model is trained in multiple rounds, which can be specified by user (the default value for iterations is 4). In each round, first the parameters of the model are learned using a stochastic gradient descent approach. The training is done in iterations until all the parameters of the model have changed by less than 0.0001 compared to the previous iteration. Training also will stop when reaching 1000 iterations to handle oscillating situations around the optimum values. This number is high enough to give the model enough time to be trained well and has been chosen by the developers based on running the model on multiple Hi-C datasets (25 samples with 2–4 different resolutions). Next, a P-value is calculated for each interaction using the trained model and the real interactions are identified based on a fixed user-defined P-value as significance limit. The read count of these real interactions is substituted with their expected read count for calculating the total number of reads for each bin, as if they were not read what would their read count be, to remove the bias of having real interactions from bias factors of the bins. They are also completely ignored in the training of the model’s parameters. This is another main difference between MaxHiC and other models.

### An automatic way of removing the effect of noisy data

Typically, a high percentage of interacting fragments in Hi-C have only 1 read between; for example, the typical percentage can be above 85% for a resolution of 5Kb. End bins of many of these interactions have very low coverage which results in a very small expected value for the interaction. This can pull the value of parameters down for the benefit of themselves in the model. In order to cope with such noisy data, the expectation for read count is calculated based on Eq 6 instead of Eq 2. In this way, the maximum of expected value and a base expected read count, *base*_*e*_, is used as the final expected value. This will prevent the mentioned noisy interactions to affect greatly the parameters of the model.


$$\mu_{ij}=softmax(v_{i}\times v_{j}\times f(d_{ij}),base_{e})$$ (6)

### A negative binomial regression background model for capture Hi-C data

Capture-Hi-C is a version of Hi-C in which some regions in DNA, which are called *Baits* or *Target Regions*, are sequenced with higher depth than the others. This will result in three different types of interactions in Capture-Hi-C with different properties, Target-Target, Target-Other and Other-Other. The background model developed for Capture-Hi-C follows the same statistical model as the one developed for General-Hi-C, but in order to account for the differences between these types of interactions, three different sets of parameters are used, each for one interaction type. Each set of parameters is learned separately from the others based on its own interactions, in the process of learning the parameters of the model. In calculating the bias factors for bins, the sum of read counts cannot be used directly as different interactions correspond to different types. To remove this barrier, all the read counts of interactions are converted to their equivalent read-counts in Target-Target interaction type:

$$x_{ij}^{t2}=x_{ij}^{t1}\times \frac{\mu_{ij}^{t2}}{\mu_{ij}^{t1}}$$ (7)

In Eq 7, t_1_ and t_2_ stand for the original interaction type and the one we want to convert the read-count to, which is assumed to be Target-Target, respectively. The expected value for each interaction type is easily calculated by using the set of parameters related to that interaction type and the calculated properties of the interaction, sum of reads for bins and their genomic distance, in the expected read-count formula regardless of the original type of the interaction.

### Justification for the estimation of expected value of read count for trans chromatin interactions

In MaxHiC, distance is modelled by a function that decreases at increasing genomic distances to reach a small but constant non-zero value to account for random ligations. For trans-interactions, we use the same constant value as observed for distant cis interactions. Here two things are considered in the function modelling distance, more exactly the function modelling expected random ligations based on distance. It is a strictly decreasing function and it converges to a constant nonzero value when distance gets high. Which means it converges to a constant value when distance gets larger than a limit. In trans interactions, distance is not close so the same constant is considered in calculating the expected value.

To show that the long distance cis have similar read counts as trans, we counted average read count of long distance (≥ 100Mb) significant cis interactions (P-value < 0.001) against average read count of all significant trans interactions (P-value < 0.001). As a results, long distance significant cis interactions had a very similar average read count (21.7) as significant trans interactions (22.1). Note: the average read count of all significant cis interactions was 29.4. We also plotted distribution of number of significant long distance cis interactions against significant trans interactions. As the plot shows both groups have almost the distribution **S19 Fig**.

### Assessment of feature enrichment (ENCODE regulatory features, FANTOM5 promoters/enhancers)

#### Annotation datasets

Tissue specificity is an important aspect of many genetic diseases [40]; We therefore downloaded tissue-specific ChIP-Seq data in the form of processed peak calls for histone modifications (e.g. H3K27ac, H3K4me1, etc), CTCF binding sites, DNase hypersensitive sites, and ChromHMM predicted promoters and enhancers from the ENCODE project [19]. We used FANTOM5 active promoters and transcribed enhancers from the FANTOM5 consortium [41, 42]. FANTOM5 tissue-specific enhancers were obtained from [42].

#### Region-based analysis

To compute the enrichment of interactions overlapping an epigenetic feature, we first calculated the fraction of significant interactions with at least one end overlapping a feature. We then calculated fraction of all interactions overlapping with the feature as the genome-wide expectation. The enrichment value was then calculated by dividing the fraction of significant interactions overlapping a feature by the genome-wide expectation.


$$Enrichment\;value=\frac{fraction\;of\;significant\;interactions\;overlapping\;a\;feature\;}{fraction\;of\;all\;interactions\;overlapping\;a\;feature}$$ (8)

To calculate the enrichment P-value, we used Fisher’s Exact test in the following manner: (i) number of significant interactions overlap with the feature; (ii) number of significant interactions that do not overlap with the feature; (iii) number of all interactions overlap with the feature; (iv) number of all interactions that do not overlap with the feature. The same approach is used to calculate the P-values for other enrichment analyses in this study.

#### Pair-based analysis

To compute the enrichment of interactions that both sides of an interaction overlap with an epigenetic feature, we repeated the above analyses in which both sides of interaction overlap with the feature.

#### Capture Hi-C analysis

In the capture Hi-C analysis, to ensure our results were not affected by the strong peak signal over target regions, only target-interacting regions (“bait-to-any interactions”) are considered and signals that mapped as bait-bait interactions and those signals that mapped outside of the target-interacting regions are excluded from the analysis. To calculate the fraction of significant target-interacting regions overlapping an epigenetic feature in the capture Hi-C analysis, we first calculated the fraction of significantly target-interacting regions overlapping a feature. We then randomly selected fragments that had no interaction with the target regions (we selected exactly the same number of fragments as the number of significant target-interacting fragments). The enrichment value then calculated by dividing the fraction of significant interacting regions overlapping a feature on the average fraction of 1000 permutations. We performed the above analysis for each Hi-C background model, separately.


$$Enrichment\;value\;in\;capture\;Hi‐C\;data=\frac{fraction\;of\;significant\;interactions\;overlapping\;a\;feature\;}{average\;fraction\;of\;random\;interacting\;regions\;overlapping\;a\;feature\;in\;1000\;permutations}$$ (9)

### GWAS data analysis

GWAS SNPs for autoimmune disease, neurological/behavioural traits and kidney/liver/lung disorders were downloaded from Maurano *et al*. [29]. To compute the enrichment of interactions overlapping GWAS SNPs, we first calculated the fraction of significant interactions with at least one end containing GWAS SNPs. We then calculated fraction of all interactions overlapping GWAS SNPs as the genome-wide expectation. The enrichment value was then calculated by dividing the fraction of significant interactions overlapping GWAS SNPs by the genome-wide expectation. In the analysis of capture Hi-C library, we only considered overlapping of GWAS SNPs with target-interacting regions in both the significant target-interacting regions and the permutated target-interacting regions.

### Overlapping Hi-C interactions with eQTL pairs

We used the set of GTEx v7 eQTLs identified as significant in EBV-transformed lymphocytes from the Genotype-Tissue Expression (GTEx) Project [21]. To calculate the fraction of eQTL pairs overlapping with significant interactions, we considered those Hi-C interactions where one side of the interaction overlapped an eQTL SNP and the other side overlapped the promoter of the eQTL target gene identified by GTEx. The promoter regions are defined as 5kb± of starting point of a gene (TSS). To calculate enrichment, we divided these by the genome-wide expectation.

### CRISPRi validation

To validate P-E pairs identified in Hi-C data, an orthogonal dataset of validated enhancer-promoter pairs was taken from Nasser et al. publication [26]. CRISPR perturbations in K562 cells identified 103 non-promoter elements involved in 134 functional element-gene relationships defined as causing significant changes in gene expression in K562 cells. We then counted a) number of non-promoter elements that overlapped with significant fragments identified by each model; b) number of element-gene pairs that overlapped with significant pairs identified by each model.

### Parameters used for comparative Hi-C tools

All tools were run with their default parameters. For SIC models, we used P-value 0.001 to identify significant interactions. For loop callers, we used FDR 0.05 to identify significant loops.

## Supporting information

S1 Information In this file, we provided an instruction to run MaxHiC. (PDF)

S1 Fig Intuitive diagram showing the procedures and logic of MaxHiC. (TIFF)

S2 Fig Venn diagram of the top 100K significant interactions identified by each model in the Rao *et al*. [5] GM12878 and HMEC datasets. **a)** Venn diagram of top 100K significant interactions identified by each model in the GM12878 data at fragment size 10kb. Loop callers are highlighted by red colour. The number of identified loops for each method are 10,487 for HiCCUPS, 18,148 for Peakachu, and 7,346 for Mustache. **b)** Venn diagram of significant interactions identified by each model in the HMEC data at fragment size 10kb. We did not include Fit-Hi-C2 in this figure as the results are almost the same as for Fit-Hi-C. Loop callers are indicated in red font. The number of identified loops are 12,053 for HiCCUPS, 18,385 for Peakachu, and 9,469 for Mustache.
(TIFF)

S3 Fig Venn diagram of the top 20K significant interactions identified by each model in the Rao *et al*. GM12878 and HMEC datasets. **a)** Venn diagram of top 20K significant interactions identified by each model in the GM12878 data at fragment size 10kb. Loop callers are indicated in red font. The number of identified loops for each method are 10,487 for HiCCUPS, 18,148 for Peakachu, and 7,346 for Mustache. **b)** Venn diagram of significant interactions identified by each model in the HMEC data at fragment size 10kb. We did not include Fit-Hi-C2 in this figure as the results are almost the same as for Fit-Hi-C. Loop callers are indicated in red font. The number of identified loops are 12,053 for HiCCUPS, 18,385 for Peakachu, and 9,469 for Mustache.
(TIFF)

S4 Fig Distance distribution of read-count support of significant interactions identified by the SIC models in GM12878 and HMEC. **a)** Minimum read-count of significant interactions in the Rao *et al*. [5] GM12878 data, **b)** as in a but for HMEC, **c)** Average read-count of significant interactions in the Rao *et al*. GM12878 data and **d)** as in c but for the HMEC data. Fragment size 10kb.
(TIFF)

S5 Fig Comparisons of the number of significant interactions and average read-counts of significant interactions identified by SIC models at fragment sizes 1kb, 5kb, 10kb, 50kb. Plots as in **Fig 1**.
(TIFF)

S6 Fig Enrichment of Histone marks in the significant interactions identified by MaxHiC. The histone marks were downloaded in the peak calling format from UCSC genome browser.
(TIFF)

S7 Fig Enrichment of GM12878 specific regulatory potential in top 100K, 50K, 20K, 10K regions identified by MaxHiC and other tools. **a)** Top 100K interactions identified by each model. **b)** Top 50K interactions identified by each model. **c)** Top 20K interactions identified by each model. **d)** Top 10K interactions identified by each model.
(TIFF)

S8 Fig Enrichment of transcription factor binding sites from ENCODE ChIP-seq data on Rao *et al*. [5] GM12878 data. As the plot shows the enrichments in MaxHiC’s interactions are similar to the enrichments in the chromatin loops identified by HiCCUPS (which identified 34-fold fewer interactions) and much better than SIC models as well as two other loop callers.
(TIFF)

S9 Fig Enrichment of regulatory features in model specific interacting regions on Rao *et al*. GM12878 Hi-C data. **a)** Interacting regions identified by MaxHiC only versus interacting regions identified by Fit-Hi-C2 only. The number of MaxHiC-specific interacting regions is 39,629; and the number of Fit-Hi-C2-specific interacting regions is 516,719. **b)** Interacting regions identified by MaxHiC only versus interacting regions identified by HiCCUPS. The number of MaxHiC-specific interacting regions is 219,397; and the number of HiCCUPS-specific interacting regions is 61. P-value < 0.001 and fragment size = 10kb.
(TIFF)

S10 Fig An example of Hi-C interaction identified by MaxHiC as significant, which was not identified as a significant interaction by other tools. Both sides of the interaction have strong signals of regulatory features.
(TIFF)

S11 Fig Fraction of regulatory pairs in the top 100K and 20K significant interactions identified by each model in GM12878 and HMEC cell lines. **a)** GM12878 – 100K. **b)** HGM12878 – 20K. **c)** HMEC– 100K. **d)** HMEC– 20K.
(TIFF)

S12 Fig Hi-C interaction map on GM12878 HiC data. Heatmap showing the raw read count of interactions at 5k bins of chr21 (36000kb to 39500kb) (same region as S10 Fig in the cLoops paper [43] on the GM12878 Hi-C dataset in Rao *et al*. [5]. The heatmap is colored based on the log of read count. Significant interactions identified by **a)** MaxHiC and **b)** HiCCUPS, are shown in black. A zoomed view of one TAD is shown in **c)** and **d)**.
(TIFF)

S13 Fig Venn diagrams showing a) Overlap between the sets of interactions identified by MaxHiC and HiCCUPS on the GM12878 dataset. b) Overlap of 10kb genomic windows involved in significantly interacting regions identified (Note: for HiCCUPS only the 10kb centroids are counted). c) Similar to b, but showing overlap when the entire anchor sequence is considered for HiCCUPS.
(TIFF)

S14 Fig Micro-C data analysis. **a)** Contact map on Micro-C data from Kreitensten *et al*. [22] (chr21:36000kb-39500kb). **b)** Aggregate peak analysis (APA) of significant interactions called by MaxHiC on Micro-C data. Interactions were called at 5kb resolution using the Micro-C Hi-C dataset from Kreitensten *et al*. [22]. The analysis shows the aggregate background interaction frequency of 5 bins upstream and downstream of the significantly interacting bins. APA plots are shown for the top 5k, and 10k.
(TIFF)

S15 Fig RING1B-depleted Hi-C analysis. **a)** Hi-C Contact map comparing significant interactions identified in WT and RING1B-/- cells at 10kb fragment size. Black pixels are significant interactions identified by MaxHiC at P-value <0.001. chr5:28.3-30Mb). **b)** Distance distribution of significant interactions identified by MaxHiC (Pvalue <0.001, 10kb bins) in RING1B-/-, and RING1B WT cells. c) Enrichment of Ring1b (left) and H3K27me3 (right) binding sites in interacting regions found i) only in the wild type cells, ii) only observed in the RING1B knockout cells and iii) those stably observed in both. Data is from [24].
(TIFF)

S16 Fig Number of CRISPRi interactions captured by each model in k562 Hi-C data. Y-axis shows number of CRISPRi interactions captured by each model and X-axis shows number of significant interactions/loops identified by each model. **a)** The total number of CRISPRi elements was 103. **b)** The total number of CRISPRi element-gene pairs was 134. CRISPRi data is from [26].
(TIFF)

S17 Fig Comparison of the four models in the analysis of capture Hi-C experiment in capture Hi-C sample GM12878. We also developed a separate version of MaxHiC to handle capture Hi-C libraries. In the capture Hi-C library, we are particularly interested to identify significant interactions that at least one end of interaction is baited (i.e., a capture region). Enrichment of GM12878 specific histone marks, putative regulatory regions and GWAS signal in regions identified by CHiCAGO, GOTHiC, Fit-Hi-C, Fit-Hi-C2, HiCDC+ and capture version of MaxHiC, on the capture Hi-C library GM12878 from Mifsud *et al*. [15]. This showed that significant target-interacting pairs identified by MaxHiC have much higher enrichment for regulatory pairs and autoimmune related GWAS SNPs.
(TIFF)

S18 Fig Comparisons of the number of significant interactions and average read-counts of significant interactions identified by SIC models at fragment sizes 1kb, 5kb, 10kb, 50kb capture Hi-C sample GM12878. Plots as in Fig 1. Capture Hi-C data was obtained from Mifsud *et al*. [15].
(TIFF)

S19 Fig Distribution of number of significant long distance cis interactions (>100Mb) against significant trans interactions in Rao *et al*. [5], GM12878 Hi-C sample. All significant cis interactions identified by MaxHiC at P-value < 0.001 that had distance more than 100Mb were categorized as long-distance cis interactions. All trans interactions with P-value < 0.001 were identified as significant trans interactions. Long distance significant cis interactions had a very similar average read count (21.7) as significant trans interactions (22.1).
(TIFF)

S1 Table Details of publicly available Hi-C samples used in this study. (XLSX)

S2 Table The statistical summaries of significant interactions identified by MaxHiC, GOTHiC, CHiCAGO, Fit-Hi-C, Fit-Hi-C2, HiCDC+, HiCCUPS, Peakachu, and Mustache on the Rao HMEC Hi-C library at fragment sizes 1kb, 5kb and 10kb. (XLSX)

S3 Table Enrichment of transcription factor binding sites in the significant interactions identified by SIC models on the Rao GM Hi-C library at fragment sizes 10kb. (XLSX)

S4 Table Details of autoimmune and breast cancer traits used in this study for GWAS analysis. (XLSX)

S5 Table The statistical summaries of significant interactions identified by MaxHiC, GOTHiC, CHiCAGO, Fit-Hi-C, Fit-Hi-C2, and HiCDC+ on the Mifsud GM12878 capture Hi-C library at fragments 5kb and 10kb. As the table shows, the average read count of significant interactions identified by MaxHiC are much higher than those identified by CHiCAGO, GOTHiC, Fit-Hi-C, Fit-HiC2 and HiCDC+.
(XLSX)

## References

[1] Carty M, Zamparo L, Sahin M, Gonzalez A, Pelossof R, Elemento O, et al. An integrated model for detecting significant chromatin interactions from high-resolution Hi-C data. Nat Commun. 2017;8:15454. doi: 10.1038/ncomms15454 28513628 PMC5442359

[2] Ay F, Bailey TL, Noble WS. Statistical confidence estimation for Hi-C data reveals regulatory chromatin contacts. Genome Res. 2014;24(6):999–1011. doi: 10.1101/gr.160374.113 24501021 PMC4032863

[3] Cairns J, Freire-Pritchett P, Wingett SW, Varnai C, Dimond A, Plagnol V, et al. CHiCAGO: robust detection of DNA looping interactions in Capture Hi-C data. Genome Biol. 2016;17(1):127. doi: 10.1186/s13059-016-0992-2 27306882 PMC4908757

[4] Mifsud B, Martincorena I, Darbo E, Sugar R, Schoenfelder S, Fraser P, et al. GOTHiC, a probabilistic model to resolve complex biases and to identify real interactions in Hi-C data. PLoS One. 2017;12(4):e0174744. doi: 10.1371/journal.pone.0174744 28379994 PMC5381888

[5] Rao SS, Huntley MH, Durand NC, Stamenova EK, Bochkov ID, Robinson JT, et al. A 3D map of the human genome at kilobase resolution reveals principles of chromatin looping. Cell. 2014;159(7):1665–80. doi: 10.1016/j.cell.2014.11.021 25497547 PMC5635824

[6] Bonev B, & Cavalli G. Organization and function of the 3D genome. Nature Reviews Genetics. 2016;17(11):661–78. doi: 10.1038/nrg.2016.112 27739532

[7] Salameh TJ, Xiaotao Wang, Fan Song, Bo Zhang, Wright Sage M., Khunsriraksakul Chachrit, Ruan Yijun, and Yue Feng. A supervised learning framework for chromatin loop detection in genome-wide contact map. Nature communications. 2020;11(1):1–12. 10.1038/s41467-020-17239-9 PMC7347923 32647330

[8] Ardakany AR, Gezer Halil Tuvan, Lonardi Stefano, and Ay Ferhat. Mustache: multi-scale detection of chromatin loops from Hi-C and Micro-C maps using scale-space representation. Genome biology. 2020;21(1):1–17. 10.1186/s13059-020-02167-0 PMC7528378 32998764

[9] Kaul A, Bhattacharyya S, Ay F. Identifying statistically significant chromatin contacts from Hi-C data with FitHiC2. Nat Protoc. 2020;15(3):991–1012. doi: 10.1038/s41596-019-0273-0 31980751 PMC7451401

[10] Anders S, Huber W. Differential expression analysis for sequence count data. Genome Biol. 2010;11(10):R106. doi: 10.1186/gb-2010-11-10-r106 20979621 PMC3218662

[11] Robinson MD, McCarthy DJ, Smyth GK. edgeR: a Bioconductor package for differential expression analysis of digital gene expression data. Bioinformatics. 2010;26(1):139–40. doi: 10.1093/bioinformatics/btp616 19910308 PMC2796818

[12] Sexton T, Yaffe E, Kenigsberg E, Bantignies F, Leblanc B, Hoichman M, et al. Three-dimensional folding and functional organization principles of the Drosophila genome. Cell. 2012;148(3):458–72. doi: 10.1016/j.cell.2012.01.010 22265598

[13] Kingma DP, Ba J. Adam: A method for stochastic optimization. arXiv preprint. 2014;arXiv:1412.6980.

[14] Sahin M, Wong W., Zhan Y., Van Deynze K., Koche R., & Leslie C. S. HiC-DC+ enables systematic 3D interaction calls and differential analysis for Hi-C and HiChIP. Nature communications. 2021;12(1):1–11. 10.1038/s41467-021-23749-x PMC8184932 34099725

[15] Mifsud B, Tavares-Cadete F, Young AN, Sugar R, Schoenfelder S, Ferreira L, et al. Mapping long-range promoter contacts in human cells with high-resolution capture Hi-C. Nat Genet. 2015;47(6):598–606. doi: 10.1038/ng.3286 25938943

[16] Roadmap Epigenomics C, Kundaje A, Meuleman W, Ernst J, Bilenky M, Yen A, et al. Integrative analysis of 111 reference human epigenomes. Nature. 2015;518(7539):317–30. doi: 10.1038/nature14248 25693563 PMC4530010

[17] Ernst J, Kellis M. Discovery and characterization of chromatin states for systematic annotation of the human genome. Nat Biotechnol. 2010;28(8):817–25. doi: 10.1038/nbt.1662 20657582 PMC2919626

[18] Ernst J, Kheradpour P, Mikkelsen TS, Shoresh N, Ward LD, Epstein CB, et al. Mapping and analysis of chromatin state dynamics in nine human cell types. Nature. 2011;473(7345):43–9. doi: 10.1038/nature09906 21441907 PMC3088773

[19] Consortium EP. An integrated encyclopedia of DNA elements in the human genome. Nature. 2012;489(7414):57–74. doi: 10.1038/nature11247 22955616 PMC3439153

[20] Hoffman MM, Ernst J, Wilder SP, Kundaje A, Harris RS, Libbrecht M, et al. Integrative annotation of chromatin elements from ENCODE data. Nucleic Acids Res. 2013;41(2):827–41. doi: 10.1093/nar/gks1284 23221638 PMC3553955

[21] Consortium G. Human genomics. The Genotype-Tissue Expression (GTEx) pilot analysis: multitissue gene regulation in humans. Science. 2015;348(6235):648–60. doi: 10.1126/science.1262110 25954001 PMC4547484

[22] Krietenstein N, Abraham Sameer, Venev Sergey V., Abdennur Nezar, Gibcus Johan, Tsung-Han S., Parsi Krishna Mohan et al. Ultrastructural details of mammalian chromosome architecture. Molecular Cell. 2020;78(3):554–65. doi: 10.1016/j.molcel.2020.03.003 32213324 PMC7222625

[23] Haarhuis JH, Robin H. van der Weide, Blomen cent A., Yáñez-Cuna J. Omar, Amendola Mario, van Ruiten Marjon S., Krijger Peter HL et al. The cohesin release factor WAPL restricts chromatin loop extension. Cell. 2017;169(4):693–707. doi: 10.1016/j.cell.2017.04.013 28475897 PMC5422210

[24] Boyle S, Ilya M. Flyamer, Williamson Iain, Sengupta Dipta, Bickmore Wendy A., and Robert S. Illingworth A central role for canonical PRC1 in shaping the 3D nuclear landscape. Genes & Development. 2020;34(13–14):931–49. 32439634 10.1101/gad.336487.120 PMC7328521

[25] Illingworth RS, Moffat M., Mann A. R., Hunter C. J, Pradeepa M. M., Adams I. R., & Bickmore W. A. The E3 ubiquitin ligase activity of RING1B is not essential for early mouse development. Genes & development. 2015;29 (18):1897–902. 26385961 10.1101/gad.268151.115 PMC4579347

[26] Nasser J, Bergman D. T., Fulco C. P., Guckelberger P., Doughty B. R, Patwardhan T. A.,… & Engreitz J. M. Genome-wide enhancer maps link risk variants to disease genes. Nature. 2021;593(7858):238–43. doi: 10.1038/s41586-021-03446-x 33828297 PMC9153265

[27] Forrester WC, Epner E, Driscoll MC, Enver T, Brice M, Papayannopoulou T, et al. A deletion of the human beta-globin locus activation region causes a major alteration in chromatin structure and replication across the entire beta-globin locus. Genes Dev. 1990;4(10):1637–49. doi: 10.1101/gad.4.10.1637 2249769

[28] Lettice LA, Heaney SJ, Purdie LA, Li L, de Beer P, Oostra BA, et al. A long-range Shh enhancer regulates expression in the developing limb and fin and is associated with preaxial polydactyly. Hum Mol Genet. 2003;12(14):1725–35. doi: 10.1093/hmg/ddg180 12837695

[29] Maurano MT, Humbert R, Rynes E, Thurman RE, Haugen E, Wang H, et al. Systematic localization of common disease-associated variation in regulatory DNA. Science. 2012;337(6099):1190–5. doi: 10.1126/science.1222794 22955828 PMC3771521

[30] Thurman RE, Rynes E, Humbert R, Vierstra J, Maurano MT, Haugen E, et al. The accessible chromatin landscape of the human genome. Nature. 2012;489(7414):75–82. doi: 10.1038/nature11232 22955617 PMC3721348

[31] Roy S, Siahpirani AF, Chasman D, Knaack S, Ay F, Stewart R, et al. A predictive modeling approach for cell line-specific long-range regulatory interactions. Nucleic Acids Res. 2015;43(18):8694–712. doi: 10.1093/nar/gkv865 26338778 PMC4605315

[32] Whalen S, Truty RM, Pollard KS. Enhancer-promoter interactions are encoded by complex genomic signatures on looping chromatin. Nat Genet. 2016;48(5):488–96. doi: 10.1038/ng.3539 27064255 PMC4910881

[33] Bonetti A, Agostini F, Suzuki AM, Hashimoto K, Pascarella G, Gimenez J, et al. RADICL-seq identifies general and cell type-specific principles of genome-wide RNA-chromatin interactions. Nat Commun. 2020;11(1):1018. doi: 10.1038/s41467-020-14337-6 32094342 PMC7039879

[34] Li X, Zhou B, Chen L, Gou LT, Li H, Fu XD. GRID-seq reveals the global RNA-chromatin interactome. Nat Biotechnol. 2017;35(10):940–50. doi: 10.1038/nbt.3968 28922346 PMC5953555

[35] Dixon JR, Selvaraj S, Yue F, Kim A, Li Y, Shen Y, et al. Topological domains in mammalian genomes identified by analysis of chromatin interactions. Nature. 2012;485(7398):376–80. doi: 10.1038/nature11082 22495300 PMC3356448

[36] Wingett S, Ewels P, Furlan-Magaril M, Nagano T, Schoenfelder S, Fraser P, et al. HiCUP: pipeline for mapping and processing Hi-C data. F1000Res. 2015;4:1310. doi: 10.12688/f1000research.7334.1 26835000 PMC4706059

[37] Servant N, Varoquaux N, Lajoie BR, Viara E, Chen CJ, Vert JP, et al. HiC-Pro: an optimized and flexible pipeline for Hi-C data processing. Genome Biol. 2015;16:259. doi: 10.1186/s13059-015-0831-x 26619908 PMC4665391

[38] Khakmardan S, Rezvani M., Pouyan A. A., Fateh M., & Alinejad-Rokny H. MHiC, an integrated user-friendly tool for the identification and visualization of significant interactions in Hi-C data. BMC genomics. 2020;21(1):1–10. doi: 10.1186/s12864-020-6636-7 32164554 PMC7068949

[39] Lieberman-Aiden E, van Berkum NL, Williams L, Imakaev M, Ragoczy T, Telling A, et al. Comprehensive mapping of long-range interactions reveals folding principles of the human genome. Science. 2009;326(5950):289–93. doi: 10.1126/science.1181369 19815776 PMC2858594

[40] Afrasiabi A, Keane J. T., Heng J. I. T., Palmer E. E., Lovell N. H., & Alinejad-Rokny H. Quantitative neurogenetics: applications in understanding disease. Biochemical Society Transactions. 2021;49(4):1621–31. doi: 10.1042/BST20200732 34282824

[41] Forrest AR, Kawaji H, Rehli M, Baillie JK, de Hoon MJ, Haberle V, et al. A promoter-level mammalian expression atlas. Nature. 2014;507(7493):462–70. doi: 10.1038/nature13182 24670764 PMC4529748

[42] Hon CC, Ramilowski JA, Harshbarger J, Bertin N, Rackham OJ, Gough J, et al. An atlas of human long non-coding RNAs with accurate 5’ ends. Nature. 2017;543(7644):199–204. doi: 10.1038/nature21374 28241135 PMC6857182

[43] Cao Y, Chen Zhaoxiong, Chen Xingwei, Ai Daosheng, Chen Guoyu, McDermott Joseph, Huang Yi, Guo Xiaoxiao, and Han Jing-Dong J. Accurate loop calling for 3D genomic data with cLoops. Bioinformatics. 2020;36(3):666–75. doi: 10.1093/bioinformatics/btz651 31504161

