# Correction: MaxHiC: A robust background correction model to identify biologically relevant chromatin interactions in Hi-C and capture Hi-C experiments

**DOI:** 10.1371/journal.pcbi.1010515

**Published:** 2022-09-09

**Authors:** Hamid Alinejad-Rokny, Rassa Ghavami Modegh, Hamid R. Rabiee, Ehsan Ramezani Sarbandi, Narges Rezaie, Kin Tung Tam, Alistair R. R. Forrest

The Data Availability statement is incomplete. The complete Data Availability statement is:

HAR is funded by a UNSW Scientia Program Fellowship and the Australian Research Council Discovery Early Career Researcher Award (DECRA) under grant DE220101210. HAR was also supported by a WA Department of Health Near-Miss Merit Award to HAR.

ARRF is supported by an Australian National Health and Medical Research Council Fellowship APP1154524. ARRF was also supported by funds raised by the MACA Ride to Conquer Cancer and a Senior Cancer Research Fellowship from the Cancer Research Trust.

HRR and RG were supported by IRN National Science Foundation (INSF), Grant No. 96006077.

This work was funded by an Australian Research Council Discovery Project grant to ARRF (DP160101960). The funders had no role in study design, data collection and analysis, decision to publish, or preparation of the manuscript.
