# Supplementary material for: MaxHiC: A robust background correction model to identify biologically relevant chromatin interactions in Hi-C and capture Hi-C experiments
Source: PLoS Comput Biol. 2022 Jun 24;18(6):e1010241. doi: 10.1371/journal.pcbi.1010241 (PMC9262194; doi:10.1371/journal.pcbi.1010241)
Supplement: S1 Information — (PDF) [file pcbi.1010241.s001.pdf]

03 Jan 2022

Contents

MaxHiC 2
Installation 2
Requirements 2
About MaxHiC 3
  Introduction 3
  Training Procedure 3
  Capture Model 3
Running MaxHiC 4
  Positional Arguments 4
  Informative Arguments 5
  Tool-Related Arguments 5
  Training-Related Arguments 5
  Capture-Model-Related Arguments 6
Files Formats 7
  Input Files 7
    Bins File 7
    Interactions File 8
    Baits File 9
  Output Files 10
    Short Output 10
    Detailed Output 11
Questions about MaxHiC 11
Reporting Issues 12
Citing MaxHiC 12
License 12

MaxHiC

MaxHiC is a background correcting model for General and Capture Hi-C experiments that assigns significance to the recorded interactions. You can become more familiar with MaxHiC by reading the paper or the short summary presented in the **About MaxHiC** section that gives you enough information to understand the tool's arguments.

Installation

You can download the package using this link. For example using wget command you can download as follows:

```
wget https://github.com/Rassa-Gvm/MaxHiC/archive/master.zip
```

Use the following command to extract the downloaded file:

```
unzip MaxHiC-master.zip -d MaxHiC
```

This will result in a MaxHiC directory in your current working directory. Alternatively you can install git and use the following command:

```
git clone https://github.com/Rassa-Gvm/MaxHiC.git
```

Now you have downloaded the latest version of MaxHiC in a directory named MaxHiC in your working directory. You may run the tool by running Main.py script in MaxHiC directory. For more help run the following command:

```
python MaxHiC/Main.py -h
```

Requirements

You will need the following packages for running MaxHiC:

- Python 3.+
- Numpy 1.14.+
- Scipy 1.1.+
- Pandas 0.24.+
- Tensorflow 1.13.+ < 2.

About MaxHiC

Introduction

MaxHiC is a background correction model for general and capture Hi-C experiments that assigns significance level to the realness of the recorded interactions based on a predictive statistical model for the read-count of random interactions, the ones observed due to the Brownian Motion of fragments in the experiment. The model works on the interactions matrix of fixed binned DNA of any resolution. It considers a negative binomial distribution for read-count of each interaction with two parameters of dispersion factor and mean. Dispersion factor is considered the same for all interactions but the mean parameter is calculated for each interaction separately and is a function of two factors: 1. Genomic distance between two interacting bins, which increases the expectation for read-count when decreased as it increases the probability of random collisions due to Brownian Motion. 2. Bias factors of the two interacting bins as different bins have different properties and different tendencies to show up in the experiment.

Training Procedure

The model is trained in iterations and in each iteration the interactions identified as significant according to user defined p-value are eliminated so the model would be trained based on insignificant interactions to be more representative of the random interactions and to avoid the biases that real interactions have e.g. their ordinal genomic distances. There are also 3 options for users to train the model based on a part of interactions not all of them, i.e. minimum distance, maximum distance, minimum read cound. All the interactions within the genomic distance of [minimum distance, maximum distance] (in the case of cis interactions) and with read count >= minimum read count (in the case of the both cis and trans interactions) would be used in training the model. Default parameters are set in a way that all interactions are used but if you think e.g. interactions with read count below some number are not even due to the experiment process and should be completely eliminated or whether you are interested in a specific distance range and you don't want the model to be fit equally well based on all possible distances, you can use these options.

Capture Model

Capture Hi-C is a version of Hi-C that some pieces of DNA are specified as *targets* or *baits* and interactions related to them are sequences with much more depth. So there would be 3 types of interactions in the experiment *bait-bait*, *bait-other*, *other-other* which have different conditions as explained so the general models cannot work well if they do not consider their differences. In MaxHiC the same model is extended to work for capture Hi-C considering these differences. In the capture version of the model, a file specifying the location of baits is also required as an input argument. The overlap between all baits and DNA bins are calculated and bins are flagged as bait or other based on the minimum required length of overlap with bait pieces. The minimum

required overlap can be specified by two options, ratio limit which is the minimum ratio w.r.t. the length of the bin and length limit which is the absolute required length of overlap between bins and bait pieces. There is also an additional option named bait overhang which specifies the extra pieces also assumed as bait from the two ends of the original bait pieces.

Running MaxHiC

You can run MaxHiC with the following command:

```
python [Dir_to_MaxHiC]/Main.py [Arguments] base_directory save_directory
```

In which [Dir_to_MaxHiC] must be filled with the directory to the MaxHiC folder you created in the Installation section. The full command is as follows:

```
python Main.py [-h] [-d device] [-t Threads_number] [-s silent_model]
        [-do detailed_output] [-pvl significance_limit]
        [-r Training_rounds] [-rs Replacing_significants]
        [-mind Minimum_distance] [-maxd Maximum_distance]
        [-minr Minimum_read_count] [-c run_capture]
        [-brl bait_ratio_limit] [-bll bait_length_limit]
        [-bo bait_overhangs] [-bd baits_directory] [-v]
        base_directory save_directory
```

In the preceding sections these arguments are explained precisely. It's important to note that you don't need to be an expert to tune MaxHiC's parameters. You can leave them at their default values and it would work fine for any data of any resolution. These parameters are added to give you more options to use the model in the way you want. You can become more familiar with their meaning by reading **About MaxHiC** section.

Positional Arguments

base_directory
Description: This must be replaced with the directory containing the raw data you want to analyze. It should have a .matrix file containing information about interactions and a .bed file containing information about bins. The formats of **Bins File** and **Interactions File** are explained in the **Files Formats** section.

save_directory
Description: The directory to save the results in. Output files and their formats are explained in the **Files Formats** section.

Informative Arguments

-h, --help
Description: Prints a help message explaining about usage and arguments.
Accepts: No argument

-v, --version
Description: Prints tool's version.
Accepts: No argument

Tool-Related Arguments

-d, --device
Description: The device to be used for training the model. The list of available devices would be printed by -h option.
Accepts: CPU:[d]/GPU:[d], [d] must be replaced with the number of the device.
Default: CPU:0

-t, --threads
Description: The number of threads to train the model using them. Ineffective in the case of using a GPU for training the model.
Accepts: A natural number.
Default: 24

-s, --silent
Description: Whether to print messages in the middle of training the model.
Accepts: T/F
Default: True

-do, --detailed_output
Description: Whether to output fully detailed files for interactions or just with minimum required information. **Short Output** and Detailed Output formats are explained in the **Output Files** section.
Accepts: T/F
Default: F

Training-Related Arguments

-pvl, --pval_limit
Description: The p-value limit for significance of interactions.
Accepts: A real number between 0 and 1.
Default: 0.001

-r, --rounds
Description: The number of iterations used for filtering significant interactions and retraining the model. Strong recommendation: Do not use 1 as the model parameters would not be trained properly in this case.
Accepts: A natural number.
Default: 4

-rs, --replace_significants
Description: Whether significant interactions should be replaced by their expected value for calculating the bias factors of bins.
Accepts: T/F
Default: True

-mind, --min_distance
Description: Interactions with genomic distance equal to or larger than the given value would be used in training the model.
Accepts: An integer number >= 0
Default: 0

-maxd, --max_distance
Description: Interactions with genomic distance equal to or less than the given value would be used in training the model.
Accepts: An integer number >= 0 or -1 (for having no limit)
Default: -1

-minr, --min_read
Description: Interactions with read-count equal to or larger than the given value would be used in training the model.
Accepts: A natural number.
Default: 1

Capture-Model-Related Arguments

-c, --capture
Description: Whether the capture model should be run. In the case of capture data, this should be set to true.

Accepts: T/F (T for True and F for False)
Default: F

-brl, --bait_ratio_lim
Description: The minimum ratio of overlap between a bin and target regions w.r.t. the bin's length to know the bin as a target bin.
Accepts: A real number between 0 and 1
Default: 0

-bll, --bait_len_lim
Description: The minimum number of overlapping base-pairs between a bin and target regions to know the bin as a target bin.
Accepts: An integer number >= 0
Default: 1

-bo, --bait_overhangs
Description: The extra number of base-pairs from each side of a target region that will also be considered as target region.
Accepts: An integer number >= 0
Default: 0

-bd, --baits_dir *(Required for the capture model)*
Description: The directory of the file containing the list of target regions. The format is explained in the **Baits File** section.
Accepts: A valid directory

Files Formats

Input Files

Bins File

This is a file with .bed postfix in its name that contains information about the location of your bins. It must be tab delimited file without any header with the following columns:

Chromosome	Start	End	BinID

For example:

chr1	0	1000	1
chr1	1000	2000	2
...			
chr2	0	1000	
...			

Chromosome
Any format is accepted for chromosome, the only important thing is to have an identical name for all the bins in one chromosome. Bins related to one chromosome must be in a continuous range of rows.

Start
The starting location of the bin in the chromosome.

End
The end location of the bin in the chromosome. This basepair is not considered as a part of the bin as it is the starting basepair of the next bin.

BinID
This column must contain sorted unique integers from 1 to whatever number required and it must be added by one in each row.

Interactions File

This is a file with .matrix postfix in its name that contains information about the interactions. It must be a tab delimited file without any header with the following columns:

**BinID1**	**BinID2**	**Read_Count**

For example:

1	1287	1
1	8679	117
...		

BinID1
The ID of one of the ends of the interaction. It must exist in the ID column of the bins file.

BinID1
The ID of the other end of the interaction. It must exist in the ID column of the bins file.

Read_Count
The total number of ligations recorded between the fragments located in the two bins.

Baits File

This is a file containing information about the location of baits or targets in Capture Hi-C. The overlap between baits and bins are calculated and bins are flagged as bait or other_end by the given criterion in the arguments section. The file must be tab delimited with header in its 1st line containing the following columns:

Chromosome	Annotation	Start	End

For example:

Chromosome	Annotation	Start	End
chr1	Gene1Promoter	4566	6723
chr2		837123	837198
...			

Chromosome
The chromosome the bait is located in. Chromosome names must be the same names used for chromosomes in the bins file.

Annotation
The annotation of the bait. This can be left as an empty string and is not used by the tool.

Start
The starting basepair of the bait in the chromosome.

End
The last basepair of the bait in the chromosome. Unlike in bins, this basepair is considered as a part of the bait.

Output Files

In the case of general model two files, one named cis_interactions.txt and one trans_interactions.txt will be created in the given save_directory. The first one contains information about cis interactions and the other one about trans interactions. In the case of capture model 6 files will be created: bb_cis_interactions.txt, bo_cis_interactions.txt, oo_cis_interactions.txt, bb_trans_interactions.txt, bo_trans_interactions.txt, oo_trans_interactions.txt in which 'b' stands for bait and 'o' stands for other_end (non-bait) and so each file contains information about interactions of one type as specified by name.

Short Output

Output files are tab delimited with the following header in their 1st line:

bin1 ID	bin2 ID	read_count	neg_log_p_val	neg_log_q_val	exp_read_count	b1_bias	b2_bias

bin1ID
The BinID of the 1st interacting bin. In the case of Capture model for *bo* interactions, this column contains the ID of the bait.

bin2ID
The BinID of the 2nd interacting bin. In the case of Capture model for *bo* interactions, this column contains the ID of the other_end.
read_count
The total number of ligations recorded between the fragments located in the two bins.

neg_log_p_val
-ln(p-value) calculated for the interaction.

neg_log_q_val
-ln(q-value) calculated for the interaction using Benjamini-Hochberg method to correct the effect of multiple testing.

exp_read_count
The expected number of reads calculated for the interaction based on the model.

b1_bias
The bias factor calculated for bin1.

b2_bias
The bias factor calculated for bin2.

Detailed Output

bin1ID	bin1Chrom	bin1Start	bin1End	bin2ID	bin2Chrom	bin2Start	bin2End	read_count	exp_read_c	neg_ln_p_v	neg_ln_q_v	b1_bias	b2_bias	b1_read_su	b2_read_su	b1_selfless_	b2_selfless_

Apart from the columns explained in Short Output section:

bin1Chromosome
The chromosome of bin1.

bin1Start
The starting location of bin1 in the chromosome.

bin1End
The ending location of bin1 in the chromosome.

bin2Chromosome
The chromosome of bin2.

bin2Start
The starting location of bin2 in the chromosome.

bin2End
The ending location of bin2 in the chromosome.

b1_read_sum:
The total number of reads recorded for bin1 (The sum of read count of all interactions bin1 is included in).

b1_selfless_read_sum
The total number of reads recorded for bin1 aparted from its self-interaction (the case in which bin1 is the same as bin2).

b2_read_sum:
The total number of reads recorded for bin2.

b2_selfless_read_sum
The total number of reads recorded for bin2 aparted from its self-interaction.

Questions about MaxHiC

Please feel free to ask your questions about MaxHiC in its Google Groups forum. You can also post in the forum by sending an email to MaxHiC@googlegroups.com.

Reporting Issues

If you faced with any problems in using MaxHiC (exceptions, bugs, crashes) you can report it in the issues section of Github.

Citing MaxHiC

License
