# Supplementary figures and images for: MaxHiC: A robust background correction model to identify biologically relevant chromatin interactions in Hi-C and capture Hi-C experiments
Source: PLoS Comput Biol. 2022 Jun 24;18(6):e1010241. doi: 10.1371/journal.pcbi.1010241 (PMC9262194; doi:10.1371/journal.pcbi.1010241)

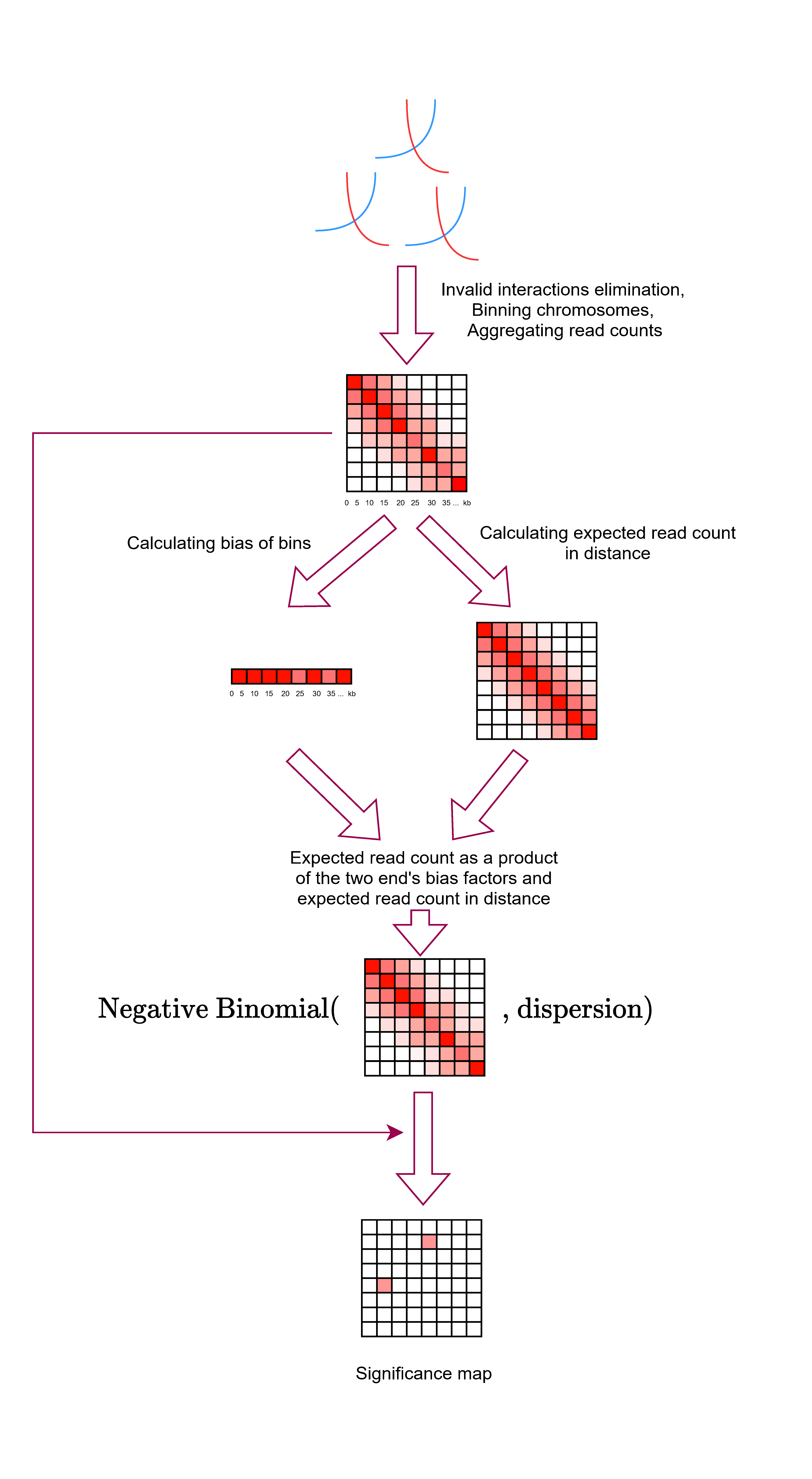

Supplement: S1 Fig — (TIFF) [file pcbi.1010241.s002.tiff]

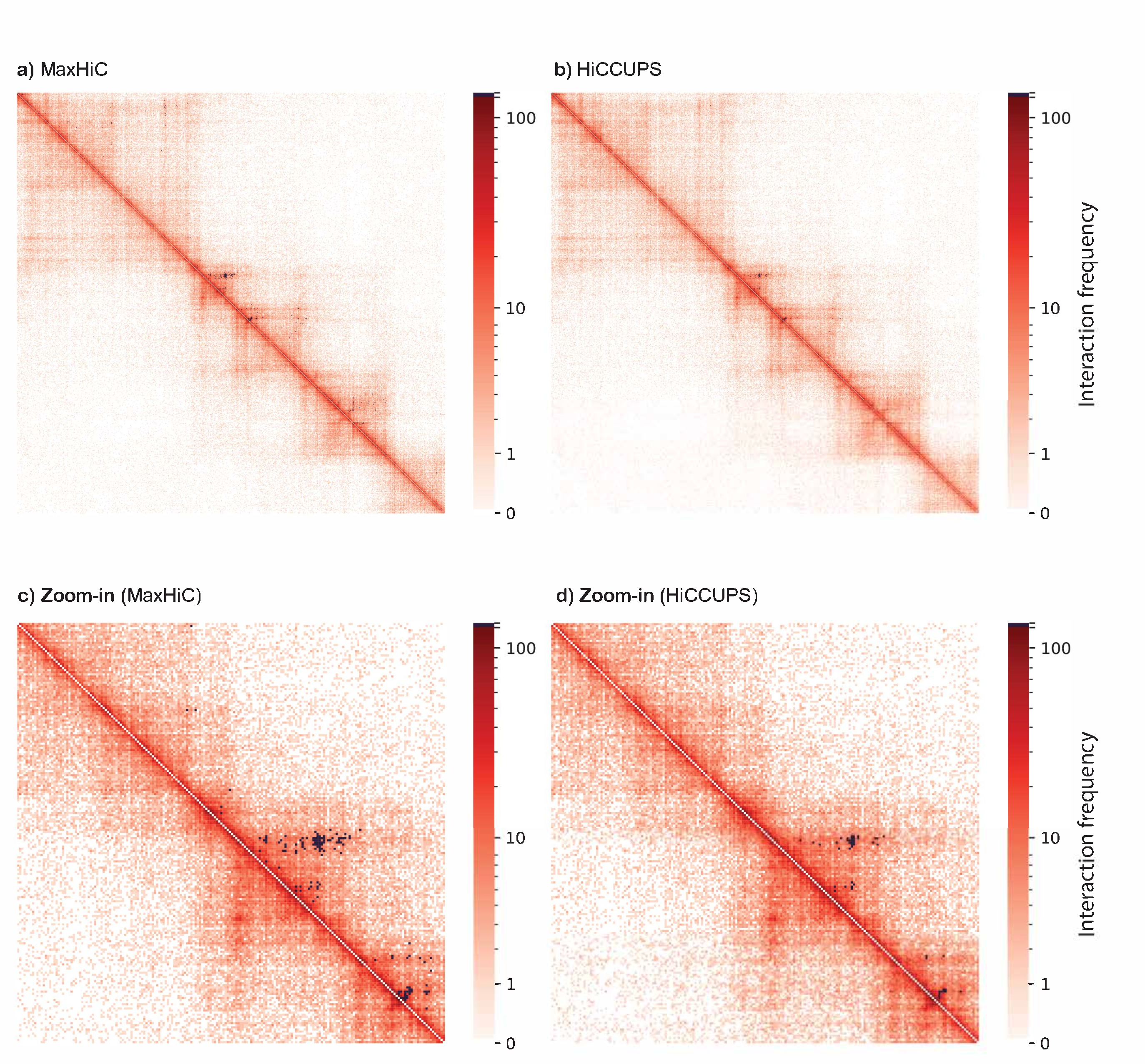

Supplement: S12 Fig — Heatmap showing the raw read count of interactions at 5k bins of chr21 (36000kb to 39500kb) (same region as S10 Fig in the cLoops paper [43] on the GM12878 Hi-C dataset in Rao et al. [5]. The heatmap is colored based on the log of read count. Significant interactions identified by a) MaxHiC and b) HiCCUPS, are shown in black. A zoomed view of one TAD is shown in c) and d). (TIFF) [file pcbi.1010241.s013.tiff]
